# 3D-QSAR and Molecular Dynamics Study of Isoxazole Derivatives to Identify the Structural Requirements for Farnesoid X Receptor (FXR) Agonists

**DOI:** 10.3390/molecules29061210

**Published:** 2024-03-08

**Authors:** Dan Yan, Yueying Yang, Hanxiao Shen, Zhen Liu, Kun Yao, Qing Liu

**Affiliations:** 1 Institute of Pharmaceutical Innovation, Hubei Province Key Laboratory of Occupational Hazard Identification and Control, School of Medicine, Wuhan University of Science and Technology, Wuhan 430065, China; 2 Institute of Cardiovascular Diseases, Hubei Province Key Laboratory of Occupational Hazard Identification and Control, School of Medicine, Wuhan University of Science and Technology, Wuhan 430065, China; 3 School of Chemical Engineering, East China University of Science and Technology, Shanghai 200237, China; 4 State Key Laboratory of Natural Medicines, Jiangsu Key Laboratory of Drug Discovery for Metabolic Diseases, Center of Drug Discovery, China Pharmaceutical University, Nanjing 210009, China

**Keywords:** farnesoid X receptor, non-alcoholic fatty liver disease, 3D-QSAR, molecular dynamics

## Abstract

The farnesoid X receptor (FXR) has been recognized as a potential drug target for the treatment of non-alcoholic fatty liver disease (NAFLD). FXR agonists benefit NAFLD by modulating bile acid synthesis and transport, lipid metabolism, inflammation, and fibrosis pathways. However, there are still great challenges involved in developing safe and effective FXR agonists. To investigate the critical factors contributing to their activity on the FXR, 3D-QSAR molecular modeling was applied to a series of isoxazole derivatives, using comparative molecular field analysis (CoMFA (q^2^ = 0.664, r^2^ = 0.960, r^2^_pred_ = 0.872)) and comparative molecular similarity indices analysis (CoMSIA (q^2^ = 0.706, r^2^ = 0.969, r^2^_pred_ = 0.866)) models, which demonstrated strong predictive ability in our study. The contour maps generated from molecular modeling showed that the presence of hydrophobicity at the R_2_ group and electronegativity group at the R_3_ group in these compounds is crucial to their agonistic activity. A molecular dynamics (MD) simulation was carried out to further understand the binding modes and interactions between the FXR and its agonists in preclinical or clinical studies. The conformational motions of loops L: H1/H2 and L: H5/H6 in FXR–ligand binding domain (LBD) were crucial to the protein stability and agonistic activity of ligands. Hydrophobic interactions were formed between residues (such as LEU287, MET290, ALA291, HIS294, and VAL297) in helix H3 and ligands. In particular, our study found that residue ARG331 participated in salt bridges, and HIS447 participated in salt bridges and hydrogen bonds with ligands; these interactions were significant to protein–ligand binding. Eight new potent FXR agonists were designed according to our results, and their activities were predicted to be better than that of the first synthetic FXR agonist, GW4064.

## 1. Introduction

Non-alcoholic fatty liver disease (NAFLD) is becoming the most common liver disease in the world, with a global prevalence of about 25%. However, currently, there are no specific drugs available for the treatment of NAFLD [1,2]. The farnesoid X receptor (FXR) is a ligand-activated transcription factor, highly expressed in the liver and intestine, which has shown promising clinical results in the treatment of non-alcoholic steatohepatitis (NASH) [3,4,5]. Numerous FXR agonists that have been developed for the treatment of NAFLD are in preclinical and clinical trials at present. The most advanced FXR agonist in clinical development is obeticholic acid [6,7], which was approved by the US Food and Drug Administration (FDA) for the treatment primary biliary cholangitis (PBC) in 2016, and which is currently being investigated in clinical evaluation for the treatment of NAFLD. Obeticholic acid improves a variety of metabolic characteristics, such as liver steatosis, liver inflammation, and fibrosis [8]. However, the use of obeticholic acid was accompanied by some side effects during clinical trials, such as pruritus and drug-induced liver injury [9,10]. Various non-steroidal synthetic FXR agonists have been developed in order to avoid these adverse reactions, and a pose filter-based ensemble learning approach was also developed to discover promising lead compounds [11]; currently, the most abundant class of non-steroidal FXR agonists is that of trisubstituted isoxazole core compounds, derived from GW4064 [12,13]. Representative isoxazole derivatives, including cilofexor, tropifexor, PX20606, and LY2562175, are undergoing clinical trials for the treatment of NASH [14]. PX20606 from Phenex was the first chemical to reach Phase I testing, where the double-bond linker was replaced by a cyclopropyl moiety [15]. Gilead Sciences compound cilofexor and Lilly compound LY2562175 are clinical candidates for NASH [16,17]. These two candidates were obtained by replacing the terminal COOH-bearing aryl with a heteroaryl and by further changing the middle linker element. To date, many GW4064 derivatives have exhibited FXR-agonistic activity both in vivo and in vitro, while the structural requirements of these isoxazole derivatives, and the relationships between their structure and activities in detail for the development of efficient FXR agonists, are unclear [18,19].

The three-dimensional quantitative structure-activity relationship (3D-QSAR) is a computational method that involves constructing models (such as comparative molecular field analysis (CoMFA) and comparative molecular similarity indices analysis (CoMSIA)) that reflect the correlations between biological activities and molecular structures, as well as investigate the interaction characteristics between ligands and proteins using various statistical methods [20,21,22,23]. The advantages of the CoMFA model are that it can consider the internal interactions of molecules as well as the interactions between molecules and their environment, providing more accurate predictions of the structure–activity relationships of molecules. The advantage of the CoMSIA model is that it can consider molecular similarity, not only predicting the activity of molecules but also predicting properties such as hydrophilicity and lipophilicity. Both the CoMFA and CoMSIA models are important methods in the field of 3D-QSAR and can play a significant role in drug design and discovery.

Molecular dynamics (MD) simulations are always employed to investigate the dynamic structure and energetic information regarding protein and ligand interactions, which are important to understanding the structure–function relationship of the complex [24]. MD simulations can reveal the interactions, binding modes, and binding abilities between proteins and ligands, helping to understand the function of proteins and the activity of ligands. In addition, MD simulations can also predict the kinetic processes of protein–ligand binding, revealing their stability and dynamics.

The combination of 3D-QSAR and MD is regarded as an effective method through which to investigate the essence of the protein–ligand interaction and to guide the drug discovery and design processes. Hence, in the present work, we explored the SARs of the GW4064 derivatives with an isoxazole moiety by performing structure–agonistic activity relationship (SAR) analysis with an MD simulation, with the aim of identifying potential candidates and designing drugs with better activities through which to target the FXR.

**Figure 1:**
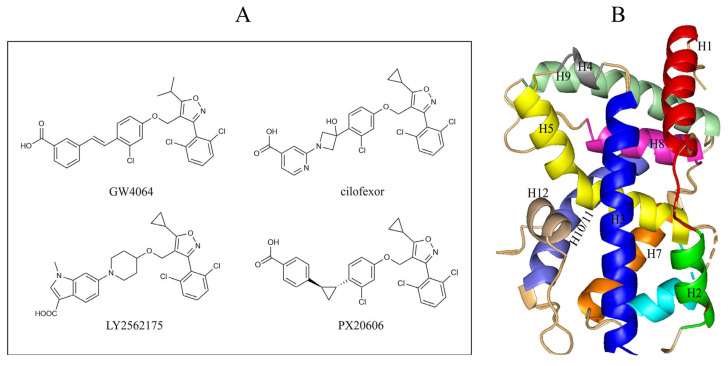
(**A**) Clinical isoxazole-based farnesoid X receptor (FXR) agonists. (**B**) The tertiary structure of the FXR (PDB ID: 6HL1).

In this study, we demonstrate an effective molecular modeling of potent FXR agonists with an isoxazole moiety using 3D-QSAR and MD simulations. To investigate the structural requirements of GW4064 derivatives as FXR agonists, CoMFA and CoMSIA models were constructed to analyze the structures and experimental activities, and the activities of GW4064, trisubstituted isoxazole core agonists with different structures in clinical studies (cilofexor, LY2562175, and PX20606) (see Figure 1A below), and the novel designed compounds were predicted. Moreover, to reveal the dynamic binding process and find the crucial residues and conformational shifts involved in ligand binding, MD simulations were conducted and compared for the complex system activities of four agonists, and the tertiary structure of the FXR is shown in Figure 1B. Root mean square deviation (RMSD), root mean square fluctuation (RMSF), hydrogen bonding, and the radius of gyration of the proteins were evaluated, with the aim of determining the flexibility and stability of the FXR when bound to different ligands. To understand how isoxazole derivatives affect the overall dynamic characteristics of the FXR, the free energy landscape (FEL), principal component analysis (PCA), and porcupine plot methods were employed to demonstrate the movement of proteins toward stable conformations within the four complex systems. Finally, the binding free energy and per-residue energy decomposition calculations for these systems were systematically analyzed, with the aim of identifying the key residues of the interaction between the FXR and ligands. Altogether, our study explored the structure–activity relationship between the GW4064-derived agonists and the FXR through combining 3D-QSAR with MD simulation, providing new insights into FXR agonist development with better safety and efficiency.

## 2. Results and Discussion

### 2.1. 3D-QSAR Model

**Figure 2:**
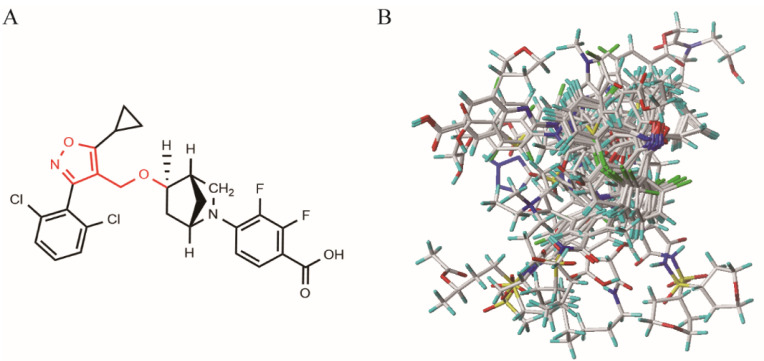
(**A**) Structure of the template molecule **190**. The common skeleton is displayed in red. (**B**) The superposition of ten high values of activity and ten low values of activity of the training set as representative compounds based on the common skeleton.

To investigate the relationships between the structures and biological activities of the isoxazole compounds, a 3D-QSAR study was conducted using CoMFA and CoMSIA. The computational statistics of the CoMFA and CoMSIA models were constructed from a training set of 203 randomly selected compounds, with pEC_50_ values ranging from 6.0 to 10.0 (Supplementary Table S2). The training set superposition, based on the common skeleton of the template molecule **190**, is shown in Figure 2, and the statistical parameters of the optimal models for the QSAR method are listed in Table 1. The best-constructed CoMFA model showed a cross-validation coefficient (q^2^) of 0.664, a non-cross-validation coefficient (r^2^) of 0.960, an F-test value of 581.48, a standard error of estimate (SEE) for cross-validation of 0.442, SEE for non-cross-validation of 0.152, SEE for the test set (SEE_pred_) of 0.261, and a prediction correlation coefficient (r^2^_pred_) of 0.872, demonstrating good predictive ability and statistical stability. The field contributions of the steric and electrostatic fields were 42.1% and 57.9%, respectively.

**Figure 3:**
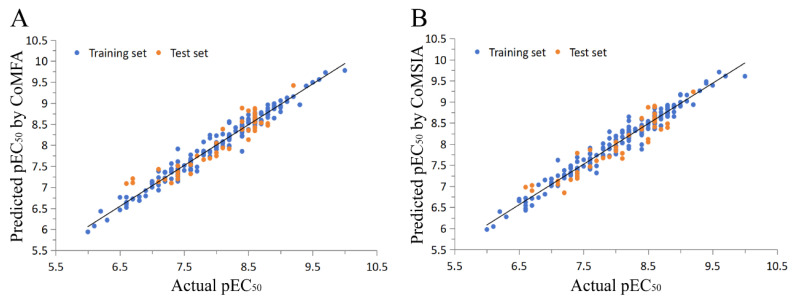
Experimental and predicted pEC_50_ values for the training and test set compounds using (**A**) the CoMFA model and (**B**) the CoMSIA model.

As compared to the CoMFA model, the CoMSIA model also calculates the hydrophobic (HF), hydrogen bond donor (HBD), and hydrogen bond acceptor (HBA) fields. For the CoMSIA study, the cross-validation coefficient was 0.706, the non-cross-validation coefficient was 0.969, the F-test value was 760.15, SEE for cross-validation was 0.413, SEE for non-cross-validation was 0.134, SEE for the test set was 0.266, and the prediction correlation coefficient was 0.866, suggesting good predictive abilities of the model (Table 1). The hydrogen bond donor field contributed the most to the molecular activity, with 30.3% (Table 1). The correlations between the predicted and experimental pEC_50_ values of the training and test sets for the CoMFA and CoMSIA models are shown in Figure 3. The activities of the four isoxazole FXR agonists under preclinical or clinical study were predicted using the QSAR model (Table 2), and they are ordered as follows: cilofexor > LY2562175 > PX20606 > GW4064. These observations are generally consistent with previous experimental results and need further verification [17,25,26,27].

### 2.2. 3D-QSAR Contour Map Analysis

**Figure 4:**
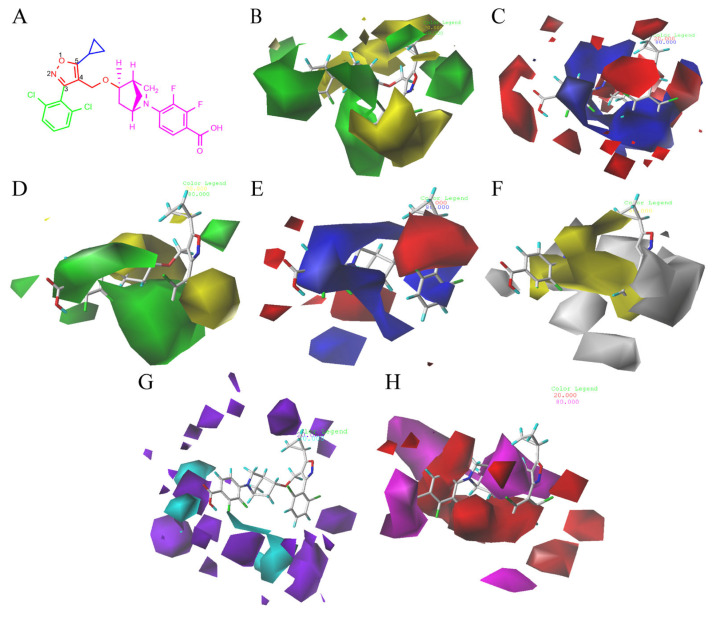
CoMFA and CoMSIA contour maps of template compound **190**. (**A**) The structure of compound **190**. CoMFA contour maps: (**B**) favorable (green) and unfavorable (yellow) steric fields, (**C**) electropositive (blue) and electronegative (red) fields. CoMSIA contour maps: (**D**) favorable (green) and unfavorable (yellow) steric fields, (**E**) electropositive (blue) and electronegative (red) fields, (**F**) favorable (yellow) and unfavorable (white) hydrophobic fields, (**G**) favorable (cyan) and unfavorable (purple) hydrogen bond donor fields, and (**H**) favorable (magenta) and unfavorable (red) hydrogen bond acceptor fields.

CoMFA and CoMSIA contour maps were generated in our study to explore the structural requirements of isoxazole-type FXR agonists. Compound **190** (Figure 4A) was chosen as the template molecule and is shown inside the field. The contours around the molecules present how the steric, electrostatic field, hydrogen bond acceptor, hydrogen bond donor, and hydrophobic interactions affect the agonistic activity. For the CoMFA model (Figure 4B), the yellow contours around the R_1_ group indicate that larger groups in this region may be detrimental to improving the agonistic activity of the compound. As can be seen from the figure, the large yellow outline around the R_2_ group suggests that the use of smaller groups in this region is beneficial for improving the agonist activity of the compound. These findings may explain the fact that the introduction of a larger group here of compounds **122** and **123** exhibited a lower pEC_50_ value. The CoMSIA steric contour map (Figure 4D) was similar to the CoMFA contour map, especially around the R_3_ group. The large green area surrounding the R_3_ group suggests that the introduction of bulky groups was favorable to FXR activity here, as in the cases of compound **87**. Previous studies consistently reported that a bulky group at the R_3_ position could be a benefit to agonistic activity [16,17,28,29].

In the 3D contour plots of the electrostatic fields in the CoMFA (Figure 4C) and CoMSIA (Figure 4E) models, the red coloring indicates that it is advantageous to introduce negatively charged functional groups, and the blue coloring indicates that it is advantageous to introduce positively charged functional groups at the indicated sites. A large blue contour was found around the benzene ring on the R_2_ group in both models, suggesting that the introduction of large electropositive substituents on the benzene ring will favor agonist activity and that the introduction of electronegativity groups is not conducive to agonist activity. As compared to compound **136**, compound **135** bearing a fluorine atom which was electronegative at the R_2_ site exhibited weaker activity. In addition, the position of the azabicyclic group and the end of the R_3_ was occupied by the red contour map, suggesting that the addition of an electronegative group here may enhance agonist activity, as in compounds **86** and **87**.

The 3D contour plot of the hydrophobic field in the CoMSIA model (Figure 4F) visualized the beneficial effects of hydrophobic groups (yellow) and hydrophilic groups (white) in the molecular structure. The large yellow contours around the R_2_ and R_3_ groups indicate that the introduction of hydrophobic groups in this position may improve biological activity. For instance, compound **126** exhibited better activity than compound **127** because of its hydrophobic group at the R_2_ site, halogen. At the same time, a white contour was located at the end of R_3_, suggesting that a hydrophilic substituent should be introduced in this position.

Figure 4G represents a contour map of the hydrogen bond donor field in the CoMSIA model, where cyan and purple contours refer to hydrogen bond donor-favored and -unfavored regions for activity, respectively. The purple contours located at the end of R_3_ indicate that the hydrogen bond donor group here may decrease biological activity. However, in the hydrogen bond donor field, the other color blocks are not explained due to their distance and small influence on the structure of the compounds.

In the contour plot of the hydrogen bond acceptor field in the CoMSIA model (Figure 4H), the favorable (magenta) and unfavorable (red) regions of the hydrogen bond acceptor field are visualized. The magenta contours at the end of R_3_ indicate that the hydrogen bond acceptor groups at these positions are important for the agonistic activity of the compound; this was exemplified by compounds **190** and **191**. The presence of a red contour around the inter- and para-positions of the benzene ring at the R_2_ site indicates that the hydrogen bond receptor group may reduce biological activity.

### 2.3. Molecular Docking

**Figure 5:**
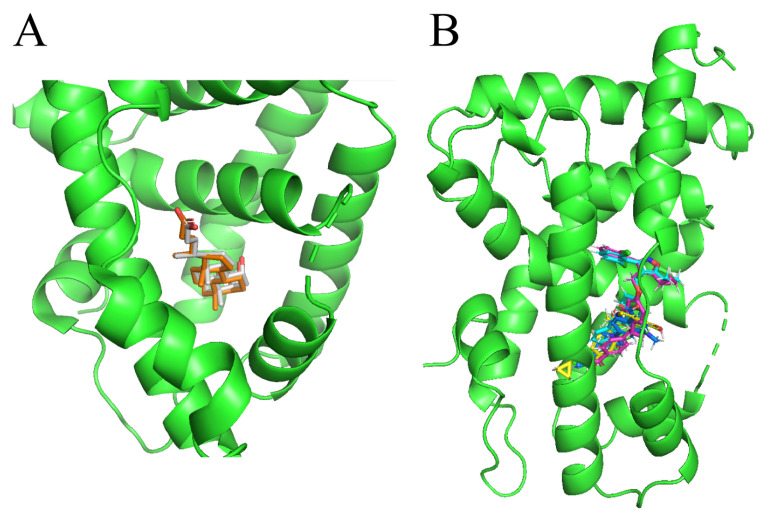
(**A**) Overlap of the docked pose and native conformation from the crystal structure (PDB ID: 6HL1). The docked pose and native confirmation are colored orange and silver, respectively. (**B**) Overlay of the binding mode of FXR and four isoxazole ligands. The green indicates FXR, yellow is used for cilofexor, cyan for GW4064, blue for LY2562175, and magenta for PX20606.

To understand the differences in the mechanisms of FXR binding and agonistic activity of isoxazoles, the binding interactions of four isoxazole compounds with FXR were studied by molecular docking. The docking software is considered reliable when it is able to generate a pose that is very close to the native conformation in the crystal structure, with the root mean square deviation (RMSD) of less than 2 Å between the docked and crystal poses [30]. The overlap of the generated docked pose and the native conformation was as shown in Figure 5A; the RMSD between these two poses is 1.96 Å, proving that the docking protocol is well established.

As shown in Figure 5B, four compounds were docked to the active site of the protein using Discovery Studio 3.0 software. The conformational affinity of the molecule for binding to the protein at that point was determined by the docking score. The site and molecule conformation with the highest LibDockScore were determined for analysis, and the obtained results are presented in Table 3. As indicated in Table 3, all isoxazoles had higher docking scores with FXR-LBD in the range of 136.612 to 141.065, suggesting that all of them can bind to FXR-LBD as agonists. The docking scores of the ligands were in the following order: LY2562175 > cilofexor > GW4064 > PX20606.

### 2.4. Molecular Dynamic Simulations

To further investigate the mechanism of the binding between ligands and the FXR, analyze their global and local structural effects, and verify the stability of the docked pose of the four medicines, an MD simulation study was performed based on the four FXR agonists (GW4064, cilofexor, LY2562175, and PX20606) under preclinical or clinical study using GROMACS (University of Groningen). All selected protein-ligand complex structures were submitted to molecular dynamics analysis. Prior to MD simulations, the ligands were integrated into complexes with proteins based on the molecular docking using Discovery Studio 3.0.

#### 2.4.1. Root Mean Square Deviation

**Figure 6:**
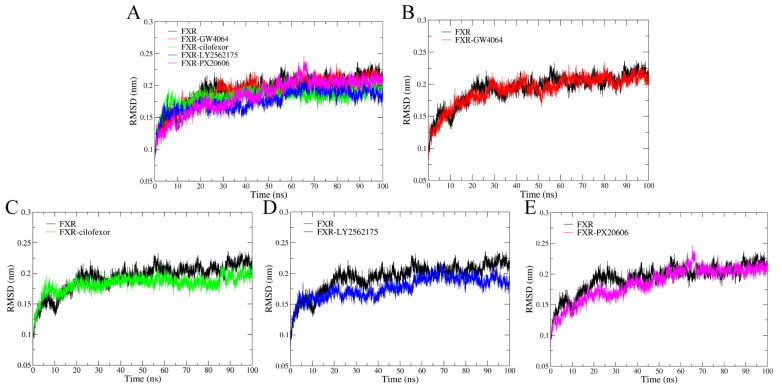
Evolution of RMSDs during 100 ns MD simulations of complexes. (**A**) The RMSDs of five systems. (**B**) FXR-GW4064 (red), (**C**) FXR-cilofexor (green), (**D**) FXR-LY2562175 (blue), and (**E**) FXR-PX20606 (magenta) are compared with the RMSD of the FXR system (black).

The root mean square deviation (RMSD) of the protein backbone was tracked throughout the MD simulations (100 ns) to assess the stability of each binding system. In general, the RMSD values of all of the systems increased rapidly within 20 ns, and then reached states of equilibrium after 65 ns (Figure 6A). The average RMSD values for the FXR, FXR-GW4064, FXR-cilofexor, FXR-LY2562175, and FXR-PX20606 systems were 0.203 nm, 0.200 nm, 0.187 nm, 0.182 nm, and 0.195 nm, respectively (Figure 6B–E). The RMSD value of the FXR was higher than that of the FXR in complex systems, indicating a higher stability of the FXR with these ligands throughout the simulations. The tendency of stability in the four complex systems can be ranked as follows: FXR-cilofexor > FXR-LY2562175 > FXR-PX20606 > FXR-GW4064. The FXR-cilofexor system is the most stable one, showing the strongest binding interaction according to the comparison.

#### 2.4.2. Root Mean Square Fluctuation

**Figure 7:**
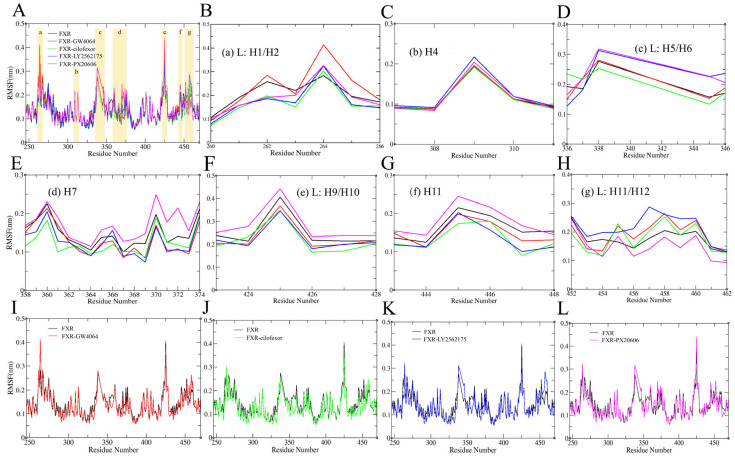
RMSFs of the backbones in all FXR systems. (**A**) The RMSFs of five FXR systems. The region RMSF plots are shown for (**B**) loop L: H1/H2, (**C**) helix H4, (**D**) the loop between helices H5 and H6 (L: H5/H6), (**E**) helix H7, (**F**) the loop between H9 and H10 (L: H9/H10), (**G**) helix H11, and (**H**) the loop between helices H11 and H12 (L: H11/H12). FXR-GW4064 (**I**), FXR-cilofexor (**J**), FXR-LY2562175 (**K**), and FXR-PX20606 (**L**) were compared with the RMSF of the FXR system. FXR-GW4064, FXR-cilofexor, FXR-LY2562175 and FXR-PX20606 systems are represented with the colors red, green, blue, and magenta, respectively.

Root mean square fluctuations (RMSFs) were measured in all systems throughout the 100 ns of MD simulation time and corresponded to the structural flexibilities of the FXR backbone atoms. The flexibilities of protein domains, and particularly loop structures close to the active site binding pocket, have earned significant attention regarding their impacts on ligand binding [31]. Binding to an FXR agonist increased the flexibility of the protein: loops L: H1/H2, L: H5/H6, L: H9/H10, and L: H11/H12, and helices H4, H7, and H11 were observed to fluctuate significantly in all systems (Figure 7A). Loop L: H1/H2 exhibited the highest flexibility in the FXR-GW4064 system, with an RMSF value reaching ~4.2 Å (Figure 7B); the other complex systems also showed high fluctuations in this region, with observations indicating that Loop L: H1/H2 significantly impacted protein flexibility. Loops L: H5/H6 and L: H9/H10 showed higher fluctuations in the FXR-LY2562175 system, with RMSF values of 3.1 Å and 2.9 Å, respectively (Figure 7D,F). Furthermore, upon comparing all of the FXR–ligand systems, helices H7 and H11 and loops L: H1/H2 and L: H9/H10 experienced lower fluctuations, but loop L: H11/H12 showed the highest fluctuation in the FXR-LY2562175 system (Figure 7B,E–H). These observations indicate that loop L: H11/H12 is an essential region in the FXR-LY2562175 system. In the FXR-PX20606 system, loops L: H5/H6 and L: H9/H10 and helices H4 and H7 showed high fluctuations. Particularly, loop L: H9/H10 showed the highest fluctuation, with an RMSF value of 4.5 Å, and helix H11 also showed a higher RMSF value, of ~2.5 Å (Figure 7C–G). It is worth noting that the complex with cilofexor showed a poor ability to increase FXR flexibility in loops L: H1/H2, L: H5/H6, and L: H9/H10 and helices H4, H7, and H11 (Figure 7B–G), which may indicate that the FXR is more dynamically stable with cilofexor. These observations are in accordance with our above-mentioned RMSD results. Consistently, ligands were reported to occupy the hydrophobic pocket of the FXR LBD and interact mainly with residues located on helices H3, H5, H7, H11, and H12 in a previous study [19].

#### 2.4.3. Hydrogen Bonding Analysis

**Figure 8:**
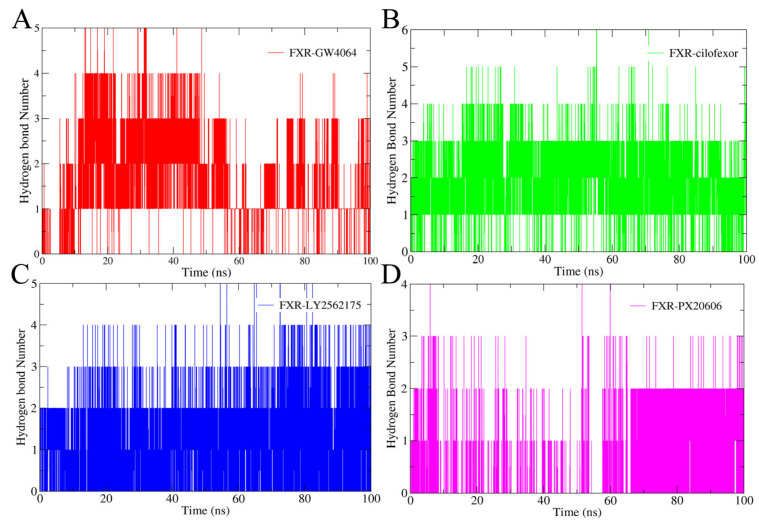
The numbers of hydrogen bonds in the FXR-GW4064 system (**A**), the FXR-cilofexor system (**B**), the FXR-LY2562175 system (**C**), and the FXR-PX20606 system (**D**). The FXR-GW4064, FXR-cilofexor, FXR-LY2562175, and FXR-PX20606 systems are represented with the colors red, green, blue, and magenta, respectively.

The hydrogen bonding patterns of the complexes over the MD simulations were analyzed (Figure 8). Hydrogen bond interactions play a crucial role in the stability of proteins [32]. Our results showed that at least two hydrogen bonds were formed between the FXR and the ligands GW4064, cilofexor, and LY2562175 (Figure 8A–C); PX20606 and FXR could also form at least two hydrogen bonds in the later period of the simulation (Figure 8D). This stable interaction suggests a crucial role of hydrogen bonding in stabilizing these four complexes. Particularly, cilofexor exhibited a higher frequency of hydrogen bonding formation with the FXR protein compared to the other ligands, suggesting that it has a greater reliance on hydrogen bonding in the interaction with FXR. The FXR-cilofexor system was consistently found to be the most stable one among the complexes, according to RMSD analysis, in our study. These findings further validate the significance of hydrogen bonding in the stabilization of FXR–ligand complexes.

#### 2.4.4. Radius of Gyration Analysis

**Figure 9:**
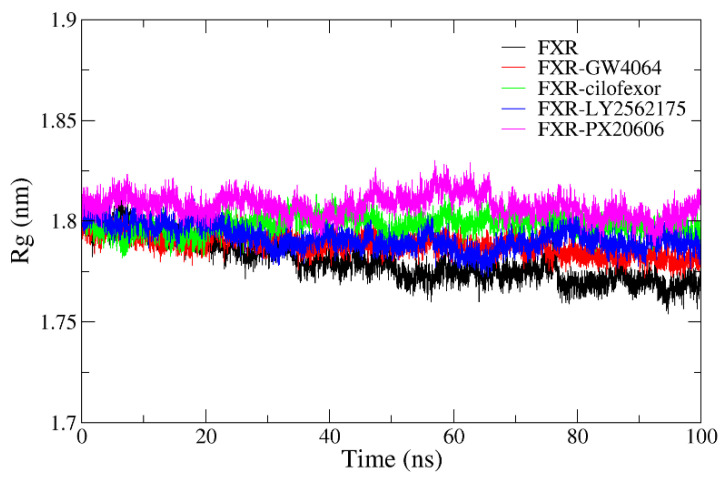
Radius of gyration (Rg) values for the FXR (black), FXR-GW4064 (red), FXR-cilofexor (green), FXR-LY2562175 (blue) and FXR-PX20606 (magenta) systems.

The radius of gyration (Rg) was employed to investigate protein structure compactness during MD simulations [33,34] (Figure 9). In our current study, the ligand–protein system was highly stable, with the Rg ranging between 1.77 and 1.83 nm. The Rg values of the four systems were observed to drop slightly, revealing more rigid complex structures during the simulation. The average Rg values of the FXR-GW4064, FXR-cilofexor, FXR-LY2562175, and FXR-PX20606 complexes were 1.788 nm, 1.798 nm, 1.792 nm, and 1.808 nm, respectively. Compared with the other complexes, the FXR-GW4064 complex showed a smaller Rg value, suggesting that it formed a more compact complex and a stronger interaction in the protein–ligand complexes. Meanwhile, the FXR system was the most compact, with an average Rg value of 1.780 nm, as compared to the other systems. This suggests that binding to the ligand increases the conformational changes to the protein and, thus, decreases protein compactness, which is consistent with the reported experimental results [35].

#### 2.4.5. Evaluation of Ligand Binding Affinities

Binding free energies, with neglected entropic contributions from the simulated systems, were calculated based on the molecular mechanics Poisson–Boltzmann surface area (MM-PBSA) method used in GROMACS (Table 4). The average values of binding free energies (∆Gbind) for the FXR with GW4064, cilofexor, LY2562175, and PX20606 were −36.71 kcal/mol, −49.83 kcal/mol, −43.46 kcal/mol, and −39.24 kcal/mol, respectively, suggesting that cilofexor possesses the strongest binding affinity with FXR, while GW4064 possesses the weakest. Smaller van der Waals force energy values represented larger contributions, and the van der Waals energies of the four complex systems (∆Evdw) were −64.69 kcal/mol, −78.87 kcal/mol, −71.11 kcal/mol, and −65.24 kcal/mol, respectively. Van der Waals energies showed much lower values than other energy terms, indicating it as the most significant contribution to the total binding free energy in our study. It is notable that the polar contributions (∆G_PB_) were positive in value, which means that they were not favorable for binding free energy.

The obtained binding free energy was in the following order FXR-cilofexor > FXR-LY2562175 > FXR-PX20606 > FXR-GW4064, which is different from the order of molecular docking score: LY2562175 > cilofexor > GW4064 > PX20606. The inconsistent results may be because molecular docking misses the protein movement and shows activity in only one conformation [36,37]. Binding free energy was employed to assess the stabilities and driving forces of intermolecular interactions [31]. The analysis of the thermodynamic driving forces of ligand–protein binding suggests that they are key components to the selection and optimization of active compounds into drug candidates [38]. It is notable that the ordering of the values involved in MM-PBSA binding affinity and the predicted activity (pEC_50_) for the four agonists was consistent in our study, demonstrating that the more stable binding of the protein to the ligand leads to the higher FXR activity [39].

#### 2.4.6. Per residue Energy Decomposition

**Figure 10:**
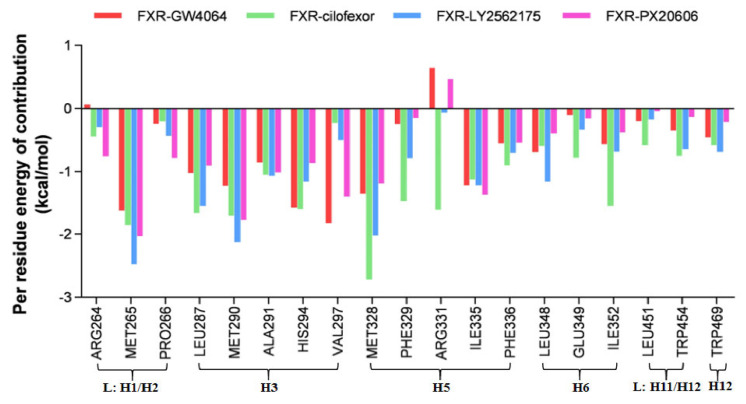
Per residue energy contributions of the binding energy ΔG_bind_ (kcal/mol) for the FXR-GW4064, FXR-cilofexor, FXR-LY2562175, and FXR-PX20606 systems. Amino acid residues with energy contributions < −1 kcal/mol are considered as key residues.

In our study, an analysis of the free energy decomposition of the residues was carried out to investigate the individual energetic contributions of each residue involved in the stabilization of protein–ligand complexes (Figure 10). The residues showing contributions of −1 kcal/mol or above were considered hot-spot amino acids and were considered to contribute significantly to the stability of the complex. According to the cutoff of the residues, MET265, MET287, MET290, HIS294, VAL297, MET328, and ILE335 showed higher energy contributions in the FXR-GW4064 system. In the case of the FXR-cilofexor system, the residues MET265, LEU287, MET290, ALA291, HIS294, MET328, PHE329, ARG331, ILE335, and ILE352 showed the greatest contributions. MET265, LEU287, MET290, ALA291, HIS294, MET328, ILE335, and LEU348 showed high energy contributions in the FXR-LY2562175 system. Similarly, for the FXR-PX20606 system, the residues MET265, LEU287, MET290, ALA291, VAL297, MET328, and ILE335 exhibited high energy contributions. Among these, residues MET265, LEU287, MET290, MET328, and ILE335 all had contributions above −1 kcal/mol, indicating their important roles in the protein–ligand binding process. Notably, residues MET328 in the FXR-cilofexor system, MET265 and MET328 in the FXR-LY2562175 system, and MET265 in the FXR-PX20606 system showed energy contributions exceeding −2 kcal/mol. In addition, the largest number of amino acids contributed less than −1 kcal/mol was shown in the FXR-cilofexor system, an observation which is consistent with the most powerful binding free energy in the FXR-cilofexor system. The residue ARG331 showed an unfavorable contribution toward the total binding free energy in the FXR-GW4064 and FXR-PX20606 systems, while it contributed strongly to that in the FXR-cilofexor system; therefore, it may be a key residue for the effect on total binding free energy.

#### 2.4.7. Protein–Ligand Interactions

**Figure 11:**
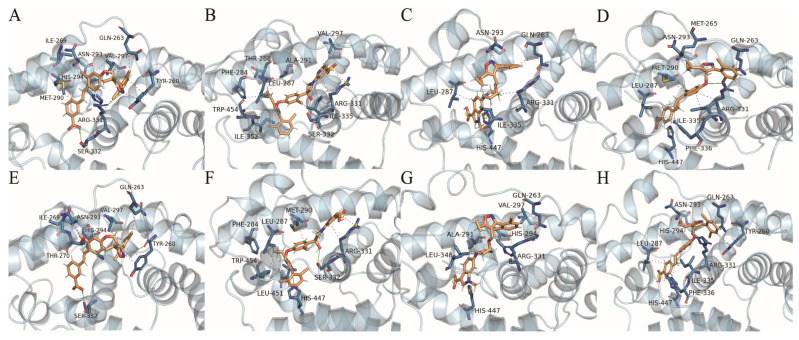
(**A**–**D**) and (**E**–**H**) show protein–ligand interactions for GW4064, cilofexor, LY2562175, and PX20606 with the FXR (blue) at 95 ns and 100 ns in the simulation, respectively. The bonds of residues and ligands are displayed as blue and orange stick structures, respectively. Hydrogen bonds are displayed as solid green lines. Salt bridges and hydrophobic interactions are displayed as orange and magenta dashes, respectively.

To further explain ligand- and structure-based conformational relationships and to reveal the protein–ligand interactions, we visualized the interactions between residues and ligands at 95 ns and 100 ns in the simulations. Protein–ligand interactions were analyzed with PLIP online, and PyMOL 2.6.0 software was used for 3D visualization (Figure 11). As shown in Table 5, the binding of FXR to four ligands mainly occurred through hydrophobic interactions, which are considered to be the most primary forces that maintain the tertiary structure of proteins [40]. Residues LEU287, MET290, ALA291, HIS294, and VAL297, which are located on the flexible helix H3 site, showed high energy contributions in the four complexes. According to our per residue energy decomposition study, these observations demonstrate the important role of helix H3 in the LBD. At 100 ns in the simulation, hydrophobic interactions were observed between GW4064 and the residues ASN293, HIS294, and VAL297 in helix H3. Hydrophobic interactions were found between cilofexor and residues PHE284, LEU287, and MET290. Also, hydrophobic interactions were found between LY2562175 and the residues ALA291, HIS294, and VAL297, and there were hydrophobic interactions between PX20606 and residues LEU287, ASN293, and HIS294. These observations further demonstrated that helix H3 (SER279~LYS304) was essential for activating the FXR. Particularly, residue MET290 formed a hydrophobic interaction with the benzoic acid group of GW4064, as well as with the chlorophenyl groups of cilofexor and PX20606. The isopropyl group of residue VAL297 interacted hydrophobically with the pyridine group of cilofexor, as well as with the dichlorobenzene in GW4064 and LY2562175. These observations demonstrated the important roles of MET290 and VAL297 in protein–ligand interactions. In addition, we also found that helix H5 (ASP312~PHE336) played an important role in protein–ligand affinity, where residues MET328, ILE335, and PHE336 made remarkable contributions to binding free energies in the four systems (Figure 10). There were hydrogen bonding interactions and salt bridges between the proteins and ligands, in addition to the hydrophobic interactions. As shown in Figure 11, the carboxyl group (–COOH) of cilofexor formed a salt bridge with the terminal amino group (–NH_2_) of ARG331 and made strongly favorable contributions to binding free energy. However, the terminal amino group (–NH_2_) of residue ARG331 formed a hydrogen bond with the methoxy group of PX20606, revealing an unfavorable contribution towards the total binding free energy. The protein–ligand interaction analysis at 95 ns simulated conformations revealed that the hydrogen bonding between the imino group (=NH) of ARG331 and the oxygen atom in the isoxazole ring of GW4064 generated an unfavorable contribution to binding free energy. ARG331 may account for the loss in binding free energies in the FXR-GW4064 and FXR-PX20606 systems, but improved binding free energy in the FXR-cilofexor system. These results suggest that ARG331 may be a key residue on helix H5; the formation of the salt bridge with the carboxyl group of ligand generates a favorable contribution to binding free energy, while the formation of the hydrogen bond with the oxygen atom of the isoxazole ring and the methoxy group of the ligand is an unfavorable factor. Moreover, although without remarkable energy contribution, HIS447 may also be a crucial amino acid residue that affects activity, as HIS447 formed a hydrogen bond with the isoxazole nitrogen of cilofexor, and formed salt bridges with the carboxyl groups of LY2562175 and PX20606. It was consistent that the activation of HIS447~TRP469 has been reported to stabilize helix H12 in conformation [19]. Therefore, our results indicate that the salt bridges (with the terminal carboxyl groups) and the hydrogen bond (with the isoxazole nitrogen) formed by residue HIS447 would be significant to the activities of derivatives.

#### 2.4.8. Conformational Flexibility in the Ligand Binding Domain of the FXR

**Figure 12:**
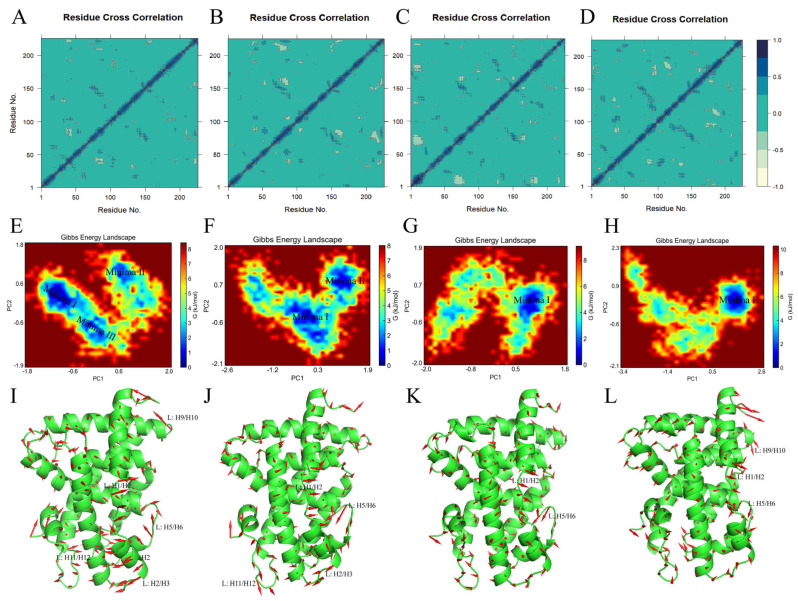
Essential dynamics of FXR–LBD systems. (**A**–**D**) Dynamic cross–correlation matrices (DCCMs) for the FXR–GW4064, FXR–cilofexor, FXR–LY2562175, and FXR–PX20606 systems. In the DCCMs, inky blue coloring represents a positive correlation, and yellowish coloring represents a negative correlation; the closer the color is to inky blue, the closer the value is to 1; the closer the color is to yellowish, the closer the value is to −1. (**E**–**H**) Free energy landscapes (FELs) of each FXR–ligand system, respective to the aforementioned order. The blue areas identify the low-energy basins (minima). (**I**–**L**) Porcupine plots of the different FXR–ligand systems, respectively. The red arrows represent the trends of the protein from its initial conformation to its most stable one. The direction of motion is represented by the arrow, and its length indicates the movement strength.

To identify protein backbone conformation shifts and monitor the effects of the four FXR agonists on the correlated motion of the FXR, an intercorrelation matrix analysis was conducted to evaluate the kinetic states of the FXR (Figure 12A–D) [41]. Protein residues were identified for correlated and anticorrelated motions using principal component analysis (PCA). The covariance matrix was diagonalized to obtain the principal components (PCs), and the eigenvalues and eigenvectors were solved to determine the directions and magnitudes of the motions. The eigenvectors show the directions of the motions, while the eigenvalues indicate their magnitudes along the direction. The output of this analysis is provided via a dynamic cross-correlation matrix (DCCM). DCCM analysis is a prominent method for analyzing the trajectory of MD simulations, with the aim of generating a matrix of cross-correlations, indicating correlations and anticorrelations between atoms during displacement or fluctuations. Correlations closer to +1 (inky blue) indicate that the residues were moving in the same direction, while correlations closer to −1 (yellowish) indicate that the residues were moving in opposite directions [42]. More yellowish blocks are shown in Figure 12B–D, suggesting more negative correlations in the motions of the residues in the FXR-cilofexor, FXR-LY2562175, and FXR-PX20606 systems and fewer negative correlations in the motions of the residues in the FXR-GW4064 system. The lowest anticorrelation in the FXR-GW4064 complex indicates less stability for the FXR-GW4064 system. These observations may help to explain why the FXR activation activity and binding free energy of GW4064 are lower than those of cilofexor, LY2562175, and PX20606.

To further investigate the effects of the four FXR agonists on the conformational space of the protein, their free energy landscapes (FELs) were analyzed. The FELs were calculated using the first two principal components, PC1 and PC2, as the coordinates with which to distinguish the conformational states of different complex systems. This method visualized the stability of the FXR and the conformational states of the four ligands by mapping out the probabilities of energy distribution for one or more variables of the protein system and identifying the low-energy basins (minima) [41,43]. The smaller and more concentrated blue areas in an FEL indicate a more stable ligand–protein complex system. Figure 12E–H depict the 2D plots of the FELs along the two primary components, PC1 versus PC2, of the FXR-GW4064, FXR-cilofexor, FXR-LY2562175, and FXR-PX20606 systems. The low- or high-energy conformations are specified with blue or red coloring in the 2D plots, respectively. Specifically, a single dominant minimum energy basin was observed for both the FXR-LY2562175 and the FXR-PX20606 complexes, while, upon the introduction of cilofexor, two major conformational regions were observed. In comparison, the conformational space of the FXR bound with GW4064 involved three energy minima, suggesting that GW4064 possesses the lowest ability to stabilize the FXR. To identify locations with high atomic fluctuations and their directionalities in the MD-simulated systems, we extracted the conformation of the deepest minima I, for each energy basin, and plotted porcupine plots using PyMOL 2.6.0 software. The basins in the FXR-GW4064 system correspond to the conformational changes in loops L: H1/H2, L: H2/H3, L: H5/H6, and L: H9/H10 and helix H2 (Figure 12I). According to the direction and magnitude of the porcupine vector, the highest fluctuation was found in loop L: H1/H2, which showed anticorrelated movement to loops L: H2/H3, L: H5/H6, and L: H9/H10 and helix H2. This indicates that the regions constituting the GW4064 binding pocket are flexible and move inward, resulting in reduced gyration and binding pocket volume, a result which is consistent with the findings previously discussed, regarding the drop in Rg. The basins in the FXR-cilofexor complex corresponded to the conformational changes in loops L: H1/H2, L: H2/H3, L: H5/H6, and L: H11/H12 (Figure 12J). In the porcupine plots, loop L: H1/H2 shows anticorrelated movement with loops L: H2/H3 and L: H5/H6, and loop L: H5/H6 showed the highest fluctuation. A slight inward movement in loop L: H11/H12 was observed in the FXR-LY2562175 system, where we observed the lowest basin (Minima I) (Figure 12K). Upon comparing the conformational changes, we found significant changes in loop L: H5/H6. The porcupine plot shows slight outward movements in loop L: H1/H2; L: H1/H2 shows anticorrelated motion with loop L: H5/H6. The FXR-PX20606 system captured slight fluctuation in loops L: H1/H2, L: H5/H6, and L: H9/H10 (Figure 12L). A slight inward movement in loop L: H1/H2 was observed. Loops L: H5/H6 and L: H9/H10 showed anticorrelated movement with L: H1/H2. In comparison, the FXR-GW4064 system showed a larger conformational deviation than the FXR-cilofexor, FXR-LY2562175, and FXR-PX20606 systems did. The region with the greatest flexibility in binding with GW4064 was L: H1/H2, which exhibited an outward movement trend, enlarging the cavity of the FXR-LBD complex and leading to a decrease in its stability. These observations may indicate that stability between loop L: H1/H2 and loop L: H5/H6 in the LBD were essential for its activation.

### 2.5. Summary of Structure–Activity Relationships

**Figure 13:**
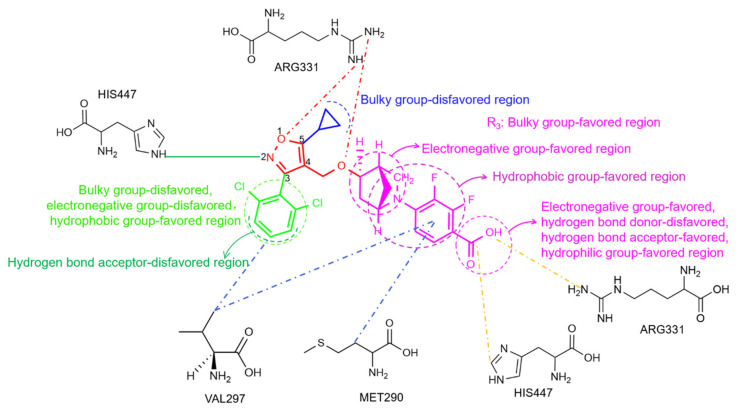
Structure–activity relationship results from the present study. The common skeleton, and its R_1_, R_2_, and R_3_ sites are shown in red, blue, green, and magenta, respectively. Unfavorable interactions, salt bridges, and hydrophobic interactions are shown as red, orange, and blue dashes, respectively. Hydrogen bonds are shown as solid green lines.

In the present work, 3D-QSAR analysis was performed using CoMFA and CoMSIA models and MD simulations, which provided a theoretical basis for the interactions between ligands and agonistic activities (Figure 13). The introduction of a hydrophobic group at the terminal position of the R_2_ site would enhance the activities of such agonists. However, the terminus of the R_2_ site was an unfavorable region for a hydrogen bond acceptor group, and the introduction of bulky and electronegative groups at the R_2_ position would result in a loss of bioactivity. A bulky group at the R_3_ site was required for improving the agonistic activity of isoxazole-type FXR agonists. Meanwhile, the introduction of a hydrogen bond acceptor group and enhancement of electronegativity at the terminal carboxyl position of R_3_ would increase the bioactivity. Alternatively, the hydrophobic group in the middle of R_3_ is also beneficial to bioactivity. Moreover, according to the MD analysis, hydrophobic interactions that formed between the ligand and the residues LEU287, MET290, ALA291, HIS294, and VAL297 were crucial for agonist activity. A protein–ligand interaction analysis demonstrated that residue MET290 formed hydrophobic interactions with benzoic acid groups, and the isopropyl group of residue VAL297 interacted hydrophobically with benzoic acid groups and with dichlorobenzene. In particular, residue HIS447, which formed salt bridges with the terminal carboxyl group and a hydrogen bond with the isoxazole nitrogen, would benefit agonistic activity. Additionally, ARG331 was a key residue of the LBD that contributed favorably to the salt bridge bound to the carboxyl group of the ligand. However, ARG331 had an unfavorable effect on the formation of hydrogen bonds with isoxazole. These results provided clues for the further modification of isoxazole compounds for better FXR agonists.

### 2.6. Design of Novel Compound

The SAR analysis provided useful information on structural features for the improvement in FXR agonist activity. Hence, we designed eight new compounds containing isoxazole cores as novel FXR agonists (compounds **246–257**) by modifying the structure of the template molecule **190**. When designing drugs, we avoided making significant changes to R_1_ and R_2_, as both of them are not suitable locations for introducing bulky groups. Instead, we focused on modifying R_3_. Compounds **247–253** were designed to increase the electronegativity and hydrophobicity of R_3_ by replacing a fluorine atom (–F) with trifluoromethyl (–CF_3_). We designed compounds **246–251** by introducing bulky groups (quinoline and naphthalene) at the R_3_ site. We also altered the hydrogen bond acceptor group–carboxyl group, as shown in compounds **249**, **252,** and **253**. Furthermore, we added strong electron-withdrawing groups at the azabicyclic group to increase the electronegativity of the compounds. Meanwhile, cyano (–CN) and nitro groups (–NO_2_) were added to compounds **246–253**. In addition, we added hydrogen bond acceptor compounds at the R_3_ group to form hydrophobic interactions and salt bridges with ARG331. The pEC_50_ values of the newly designed compounds were predicted using the CoMFA and CoMSIA models (Table 6). All of the designed compounds showed better predicted activity than compound **190** did, and these predictions are consistent with the conclusions of the SAR analysis in this study.

## 3. Materials and Methods

### 3.1. Database Selection and Biological Activity

To construct the pharmacophore model, a series of isoxazole compounds acting as FXR agonists were chosen from previously published studies (US11718619, US10793568). The experimental data obtained were all measured under the same experimental conditions, and the experimental data (EC_50_) were measured using the fluorescent resonance energy transfer (FRET) method [44]. The FRET signal is measured as the ratio of 520 nm/495 nm emission following excitation at 340 nm. We transformed the EC_50_ values into –logEC_50_ (pEC_50_) to reduce the skewed data distribution’s adverse effects and stabilize the variance [45]. Based on their structural diversity and activities, 203 compounds were randomly selected as the training set, and 42 molecules as the test set, from the 245 molecules (all sets contained template molecule) to limit the error caused by confounding factors [45]. Supplementary Table S1 lists their structures and bioactivity values, and the test set is indicated with a superscript asterisk (“*”). All compounds were obtained from PubChem database and prepared using SYBYL-X 2.0 software.

### 3.2. Molecular Optimization and Alignment

The structures of the isoxazole compounds were optimized through energy minimization by incorporating Gasteiger–Hückel charges and a Tripos force field [46,47]. The maximum iteration parameter was set to 10,000 times, the energy convergence was limited to 0.05 kcal/mol, and the other values were set to default [48]. Molecular energy optimization was performed on the molecules in the training set to obtain the lowest energy conformation for each molecule, as the quality of molecular alignment is an important factor in 3D-QSAR modeling [49]. For 3D-QSAR studies, the common skeleton was used for compound alignment. In order to maximize the comparison, the most active compound **190** was selected as the template molecule for comparison (as shown in Figure 2A).

### 3.3. Construction of the 3D-QSAR Model

The aligned training set molecules were placed in a 3D lattice box with a grid spacing of 2 Å. Then, CoMFA steric (S) and electrostatic (E) fields between the aligned compounds, and a carbon atom probe with sp^3^ hybridization and a charge of +1.0, were computed using Coulombic and Lennard–Jones potentials, respectively [50]. The CoMSIA models were created by using the same molecular alignments that were used for the CoMFA ones. The aligned compounds were placed in a 3D lattice box, with a grid spacing of 2.0 Å. For the CoMSIA model, in addition to steric and electrostatic fields, hydrophobic (H), as well as hydrogen bond donor (D) and acceptor (A), descriptors were calculated by using the same lattice box as for the CoMFA model and a sp^3^ carbon probe with a +1.0 charge [51]. Partial least squares (PLS) analysis was used to construct a linear correlation between the CoMFA/CoMSIA descriptors as independent variables and the biological activity (pEC_50_) as dependent variables [52]. The training set was cross-validated using the leave-one-out (LOO) procedure to obtain the cross-validation coefficient (q^2^) and the optimal component number (N). A regression analysis was performed with non-cross-validation to obtain the non-cross-validation coefficients (r^2^), the significance test value (F), and the standard error of estimate (SEE) [53]. The parameters q^2^, F, r^2^, and SEE are the main indices used to determine the quality of 3D-QSAR models [54,55].To ensure that the results of the 3D-QSAR model were more informative, the following requirements for these evaluation parameters must be met: q^2^ > 0.5, r^2^ > 0.9, and F > 100 [48,56].

### 3.4. External Validation of the CoMFA and CoMSIA Models

The predictive capabilities of the developed models were then evaluated using the test set of 42 compounds. After these compounds were aligned to the template, the pEC_50_ values of all compounds were predicted via the developed CoMFA and CoMSIA models. Thus, the predictive correlation coefficients (r^2^_pred_) between the predicted and experimental activities of the test set were obtained. In general, r^2^_pred_ > 0.5 is necessary for a good model [57]. The predictive correlation coefficients for the predictive performance of the PLS model were measured via the following equation [47,58,59,60]:

r^2^_pred_ = (SD − Press)/SD (1)

where SD is the sum of squared deviations from the mean of the observed activities, and Press is the predicted sum of squares, which is the sum of the squares of the difference between predicted and experimental activity.

### 3.5. Molecular Docking

Molecular docking was performed in Discovery Studio 3.0 software. The crystal structure of the FXR (PDB ID: 6HL1) with a high resolution at 1.60 Å was acquired from the Protein Data Bank (PDB) database [61]. The 3D structures of compounds were obtained from the PubChem database. Prior to molecular docking, the FXR crystal structure was cleaned, and water and original ligands were removed. The protein was pre-processed by CHARMm force field using Discovery Studio 3.0, and the ligands GW4064, cilofexor, LY2562175, and PX20606 were optimized using the MMFF force field. Then, the optimized ligands were docked into the corresponding active pockets where the original ligands were located using LibDock module, respectively. The poses obtained from docking were ranked according to the LibDock score, and the complexes with the highest LibDock score were selected as starting poses to perform MD simulation [62].

### 3.6. Molecular Dynamic Simulations

#### 3.6.1. System Preparation

GROMACS 2020.4 software was used to perform 100 ns molecular dynamics (MD) simulations for each complex.

Here, we explored five systems—no ligand in the FXR system, the FXR-GW4064 system, the FXR-cilofexor system, the FXR-LY2562175 system, and the FXR-PX20606 system—to identify the motion mechanisms of the FXR with its binding partners and determine their binding sites. A GAFF force field was employed to parameterize the ligands, while an AMBER ff99SB force field was employed for the FXR structure [63,64]. Each complex was then solvated by a dodecahedral box of TIP3P waters, with the solute 10 Å away from the water box boundary [65]. After the solvation, each system was neutralized with the addition of counter ions (NaCl), followed by the steepest descent method, in order to minimize the system every 1000 steps. Afterward, each system was heated from 0 to 300 K in 100 ps in the NVT ensemble, and then the systems were equilibrated for 100 ps at 300 K and 1 atm pressure in the NPT ensemble. Moreover, the particle mesh Ewald (PME) was utilized to derive long-range electrostatic interactions [66]. The bonds involving hydrogen were constrained by applying the SHAKE algorithm [67], allowing for a time step of about 2 fs, and periodic boundary conditions were applied in all directions. The systems were subjected to a 100 ns MD simulation at constant pressure and temperature. To assess the stability and availability of the MD, triplicate all-atom MD simulations were performed using GROMACS 2020.4 [35].

#### 3.6.2. MD Trajectory Analysis

The root mean square deviation (RMSD), root mean square fluctuation (RMSF), hydrogen bonding analysis, and radius of gyration (Rg) were used to analyze the MD trajectory. The RMSD was used to analyze global conformational dynamics and the stabilities of proteins [42]. RMSF was used to describe the fluctuation of each amino acid residue [68]. Rg was used to characterize the tightness of each system [69]. Hydrogen bonds are determined by an acceptor–donor distance of <3.5 Å and an acceptor–H–donor angle of >135° [70]. The xmgrace program (version 5.1.22) was used to generate two-dimensional (2D) plots.

#### 3.6.3. Binding Free Energy Calculations

The molecular mechanics Poisson–Boltzmann Surface Area (MM-PBSA) method was used to calculate the binding free energy of protein–ligand complexes in each system. In this study, calculations using MM-PBSA were performed to explore the binding affinity of protein–ligand complexes and systematically compare the binding free energies of each system [71]. Meanwhile, the MM-PBSA method can decompose the total binding free energy to confirm key residues that contribute significantly to binding. The last 10 ns of the MD trajectories were selected for the calculation of binding free energy (G). The specific calculation formulas are as follows [72,73]:

ΔG = G_complex_ − (G_protein_ + G_ligand_) (2)

G = E_gas_ + G_sol_ − TΔS (3)

E_gas_ = E_vdw_ + E_ele_ (4)

G_sol_ = E_PB_ + E_SA_ (5)

G_SA_ = γ·SASA (6)

where G is the total binding free energy, and G_complex_, G_protein_, and G_ligand_ are the free energies of the complex, protein, and ligand, respectively. E_gas_, the gas phase interaction energy, is evaluated as the sum of the van der Waals (E_vdw_) and electrostatic energies (E_ele_). The solvation free energy (G_sol_) is divided into polar (E_PB_) and nonpolar (E_SA_) components, in which the polar solvation energy was calculated using the Poisson–Boltzmann equation, and the nonpolar solvation energy was computed based on the solvent-accessible surface area (SASA), with γ set to the default value [74]. The configuration entropy (TΔS) was attributed to the contributions of entropy temperature and S entropy [75].

#### 3.6.4. Protein–Ligand Interaction Analysis

Ligand binding to protein is a dynamic process involving numerous structural changes in proteins and dynamic processes of ligand binding. Thus, the conformations at 95 ns and 100 ns were captured in order to explore common crucial residues, which was performed using the module gmx trjconv command. Contact residues of the complexes’ structures were analyzed using the Protein–Ligand Interaction Profiler (PLIP), and then the results of image plotting were processed using PyMOL software.

#### 3.6.5. Principal Component Analysis and Dynamic Cross-Correlation Matrix

The PCA method utilizes the covariance matrix to extract the most important functional motions from the protein dynamics coordinates. The covariance matrix includes the atomic fluctuations of each residue’s C-alpha and provides the orthogonal eigenvectors and their corresponding eigenvalues [75]. The eigenvalues represent the amplitudes of the motions, while eigenvectors describe the directions of the motions, which are then used to calculate principal components (PCs). The covariance matrix shows the displacements of the C-alpha atoms relative to their average positions; the following equation was used to calculate the covariance matrix element (C_ij_):

C_ij_ = <(x_i_ − <x_i_>)(x_j_ − <x_j_>)> (7)

where x_i_ represents the position of the ith carbon alpha atom in Cartesian coordinates, and the brackets represent the average of all structures that were sampled throughout the MD trajectory. Positive C_ij_ values show correlated motion between residues i and j. Negative values of C_ij_ show anticorrelated motion between residues i and j. The parentheses show the average of all structures sampled on the MD trajectory. The dynamic cross-correlation matrix (DCCM) was used to identify the cross-correlation shifts of the backbone atoms and to show protein backbone conformation shifts, and was performed using RStudio software (version 4.3.1) and the Bio3d application [75,76,77]. The cross-correlation coefficient value fluctuated from −1 to 1, and the resulting dynamic cross-correlation matrices displayed amino acids that have both positive and negative effects.

#### 3.6.6. Free Energy Landscape

The PCA method was employed to calculate eigenvectors and eigenvalues and their projection, along with the first two principal components. The FEL was plotted based on the first two principal components (PC1 and PC2), which were attained using the gmx sham module of GROMACS software and the xpm2txt.py tool [78]. Porcupine plotting allowed visualization of the directions and extents of primary motions exhibited by the backbone residues of different ligands and the FXR. The trajectory of each system, from its initial motion towards the lowest-energy conformation of the FEL, was analyzed in a porcupine plot analysis. All possible atomic motions were visualized as mode vector graphs. The porcupine plot was constructed using a Python script and PyMOL software.

## 4. Conclusions

We carried out a molecular modeling study on isoxazole derivatives as FXR agonists using 3D-QSAR and MD simulations. The CoMFA and CoMSIA models, with good predictive ability, were constructed for the 3D-QSAR study; the obtained contour maps depicted the structural requirements of isoxazole derivatives serving as FXR agonists, thus promoting the development of derivatives with improved agonistic activities. MD simulations of GW4064 and three isoxazole-derived FXR agonists that have entered clinical studies were investigated in our study. GW4064 exhibited the least stability in the LBD of the FXR, showing the fewest hydrogen bond interactions and the lowest binding free energy, while cilofexor demonstrated the opposite. The stability of the L: H1/H2 and L: H5/H6 loops seemed to correlate with agonistic activity in our study. A free energy decomposition analysis revealed that the residues MET265, LEU287, MET290, MET328, and ILE335 made crucial contributions to ligand binding. In addition, according to the analysis of conformational relationships between ligands and structures, the interactions in which ligands connected with residues MET290 and VAL297 in helix H3 via hydrophobic interactions would be favorable to protein–ligand affinity. Residue ARG331 of helix H5 engaged in salt bridges, and residue HIS447 participated in salt bridges and hydrogen bonding; these two residues were determined as the key residues for agonistic activity in our study. Eight new potential FXR agonists, with better predicted agonistic activity, were designed based on a structure–activity relationship analysis. In summary, these structural features contribute to the understanding of drug hotspots for the FXR and provide promising avenues for the discovery of novel FXR agonists, thus providing potential therapeutic strategies for NASH.

## Figures and Tables

**Table 1:** Statistical results of the CoMFA and CoMSIA models.

PLS Statistics	CoMFA	CoMSIA
q^2 a^	0.664	0.706
r^2 b^	0.960	0.969
SEE_cv_^c^	0.442	0.413
SEE_ncv_^d^	0.152	0.134
SEE_pred_^e^	0.261	0.266
F ^f^	581.48	760.15
N ^g^	8	8
r^2^_pred_^h^	0.872	0.866
Steric ^i^	0.421	0.103
Electrostatic ^i^	0.579	0.234
Hydrogen bond acceptor ^i^	-	0.211
Hydrogen bond donor ^i^	-	0.303
Hydrophobic ^i^	-	0.150

^a^ Cross-validation coefficient. ^b^ Non-cross-validation coefficient. ^c^ Standard error of estimate for leave-one-out cross-validation. ^d^ Standard error of estimate for non-cross-validation. ^e^ Standard error of estimate for the test set. ^f^ F-test value. ^g^ Optimal component number. ^h^ Predictive correlation coefficient. ^i^ The field contributions to the molecular activity.

**Table 2:** Predicted pEC_50_ values of isoxazole-based FXR agonists under preclinical and clinical study.

No.	Clinical Medicine	CoMFA Pred.	CoMSIA Pred.
1	cilofexor	8.748	8.427
2	LY2562175	7.855	7.814
3	PX20606	7.365	6.964
4	GW4064	7.149	6.823

**Table 3:** Docking scores of FXR and isoxazole ligands.

No.	Clinical Medicine	LibDockScore
1	cilofexor	139.386
2	LY2562175	141.065
3	PX20606	136.612
4	GW4064	137.190

**Table 4:** MM-PBSA calculations of the simulated complex.

Terms (**kcal/mol)**	FXR-GW4064 System	FXR-cilofexor System	FXR-LY2562175 System	FXR-PX20606 System
∆Eele ^a^	−3.2 ± 0.98	−13.25 ± 3.50	−8.39 ± 3.52	−3.78 ± 1.33
∆Evdw ^b^	−64.69 ± 4.35	−78.87 ± 0.56	−71.11 ± 2.80	−65.24 ± 3.92
∆E_gas_^c^	−67.89 ± 4.13	−92.11 ± 3.13	−79.51 ± 5.68	−69.02 ± 4.59
∆GPB ^d^	34.21 ± 2.00	44.91 ± 4.31	40.75 ± 4.15	33.71 ± 2.38
∆GSA ^e^	−8.02 ± 0.18	−8.32 ± 0.02	−8.53 ± 0.21	−7.85 ± 0.47
∆Gsol ^f^	26.19 ± 1.83	36.59 ± 4.30	32.22 ± 3.96	25.87 ± 1.92
∆Gbind ^g^	−36.71 ± 2.55	−49.83 ± 3.30	−43.46 ± 2.48	−39.24 ± 4.40

^a^: Electrostatic energy. ^b^: Van der Waals interaction energy. ^c^: Gas phase interaction energy. ^d^: Polar solvent effect energy. ^e^: Nonpolar solvent effect energy. ^f^: Total solvating energy. ^g^: Binding free energy.

**Table 5:** Molecular interactions of FXR–ligand complexes.

Time	Protein–Ligand Interaction	Hydrophobic Interaction	Hydrogen Bond	Salt Bridge
95 ns	FXR-GW4064	TYR260, GLN263, ILE269, MET290, ASN293, HIS294, VAL297, ARG331	ARG331, SER332	-
	FXR-cilofexor	PHE284, LEU287, THR288, ALA291, VAL297, ARG331, ILE335, ILE352, TRP454	SER332	ARG331
	FXR-LY2562175	GLN263, LEU287, ASN293, ARG331, ILE335	-	HIS447
	FXR-PX20606	GLN263, MET265, LEU287, MET290, ASN293, ARG331, ILE335, PHE336	ARG331	HIS447
100 ns	FXR-GW4064	TYR260, GLN263, ILE269, THR270, ASN293, HIS294, VAL297	TYR260, SER332	-
	FXR-cilofexor	PHE284, LEU287, MET290, ARG331, LEU451, TRP454	SER332, HIS447	ARG331
	FXR-LY2562175	GLN263, ALA291, HIS294, VAL297, ARG331, LEU348	-	HIS447
	FXR-PX20606	TYR260, GLN263, LEU287, ASN293, HIS294, ILE335, PHE336	ARG331	HIS447

**Table 6:** Chemical structures and predicted pEC50 values (Pred.) of the newly designed molecules.

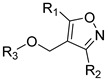					
Compound No.	R_1_	R_2_	R_3_	CoMFA Pred.	CoMSIA Pred.
**246**	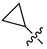	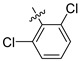	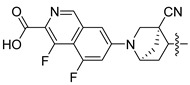	12.45	11.312
**247**	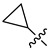	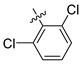	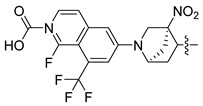	11.055	10.025
**248**	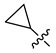	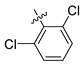	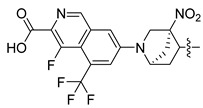	10.719	9.703
**249**	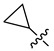	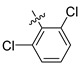	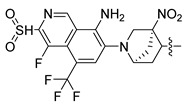	10.457	9.833
**250**	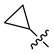	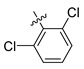	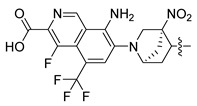	10.138	9.78
**251**	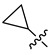	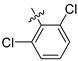	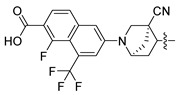	12.822	12.091
**252**	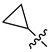	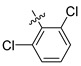	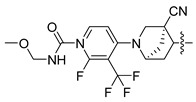	11.361	10.08
**253**	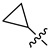	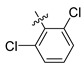	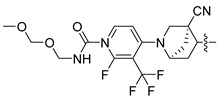	11.326	10.045

## Data Availability

Data are contained within the article and supplementary materials.

## Supplementary Materials

The following supporting information can be downloaded at: https://www.mdpi.com/article/10.3390/molecules29061210/s1, Table S1: The structures of dataset compounds; Table S2: The actual pEC50, predicted pEC50 (Pred.), and the residuals (res.) of dataset compounds.
