# Supplementary material for: 3D-QSAR and Molecular Dynamics Study of Isoxazole Derivatives to Identify the Structural Requirements for Farnesoid X Receptor (FXR) Agonists
Source: Molecules. 2024 Mar 8;29(6):1210. doi: 10.3390/molecules29061210 (PMC10974177; doi:10.3390/molecules29061210)
Supplement: Supplementary file 1 [file molecules-29-01210-s001.zip › molecules-2837380-supplementary.pdf]

Supplementary Material for

Supplementary Table S1. The structures of dataset compounds.

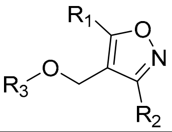

Compound no.	R_1	R_2	R_3
1	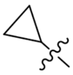	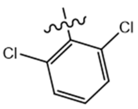	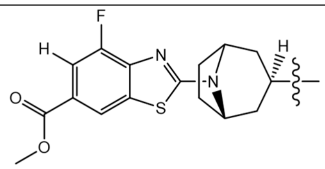
2	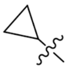	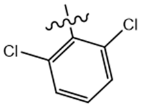	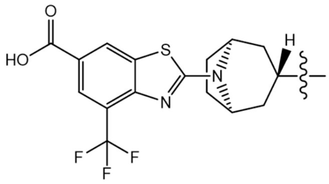
3	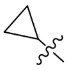	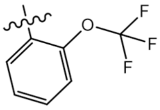	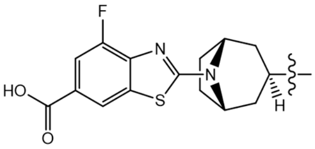
4	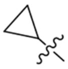	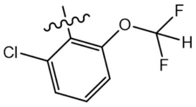	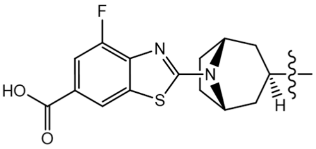
5	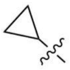	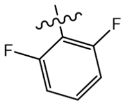	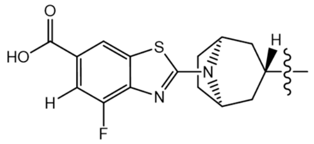
6	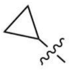	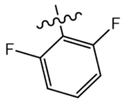	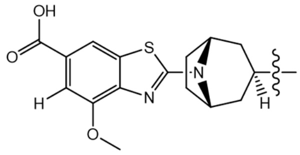
7	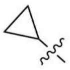	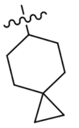	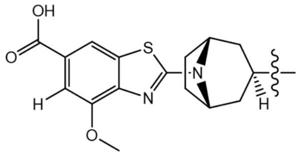
8	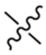	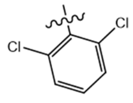	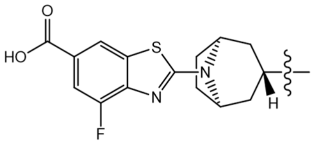
9	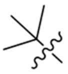	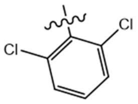	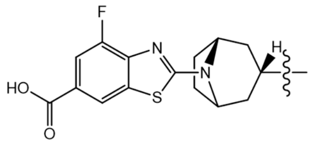

10	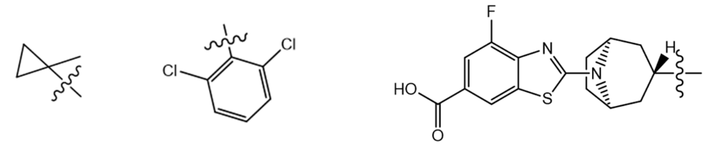
11	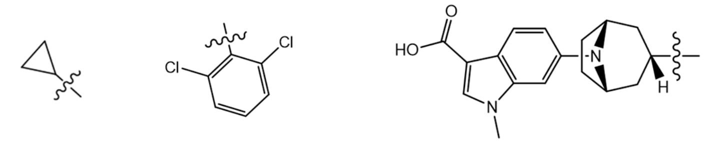
12	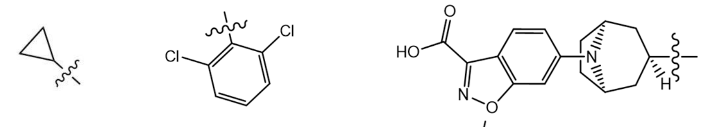
13	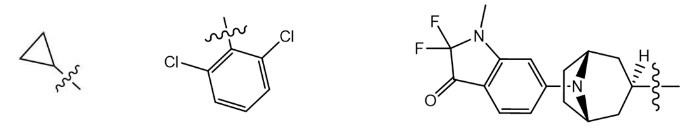
14	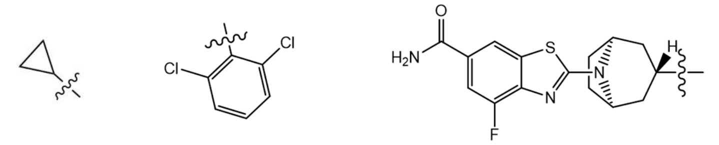
15	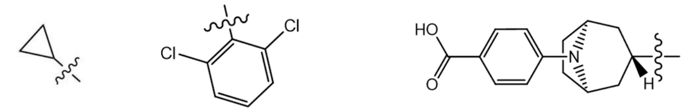
16	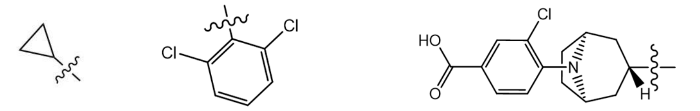
17	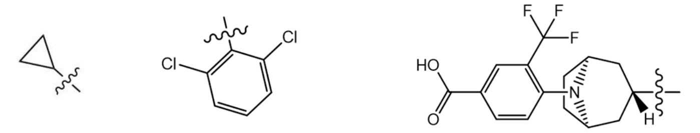
18	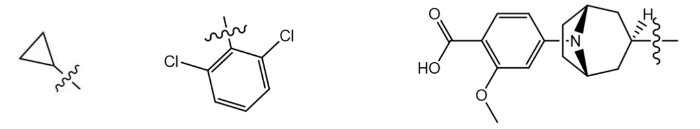
19	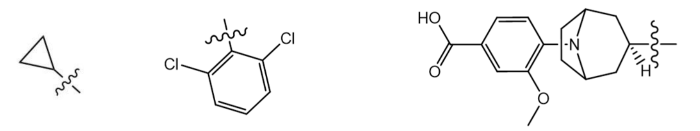
20	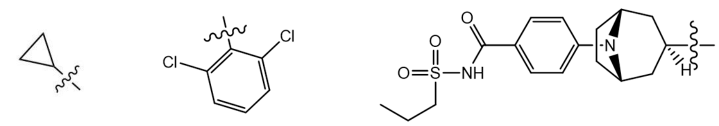
21	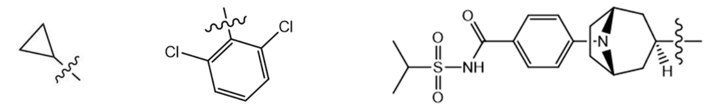
22	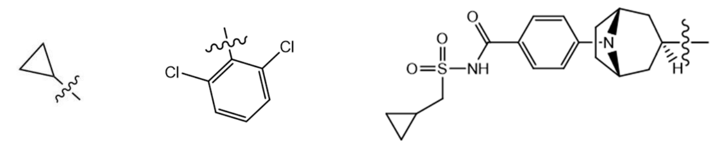

23	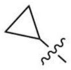	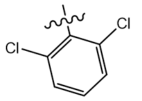	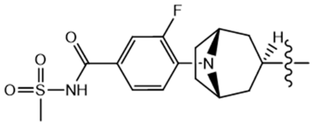
24	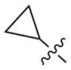	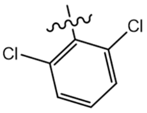	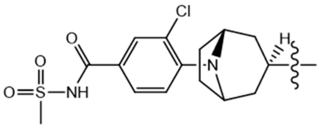
25	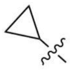	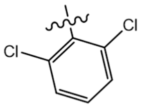	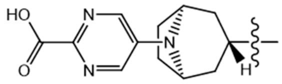
26	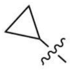	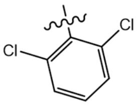	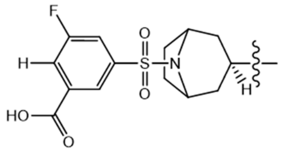
27	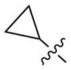	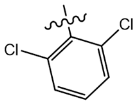	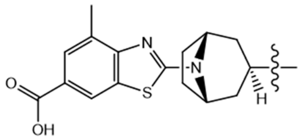
28	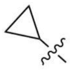	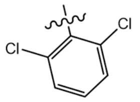	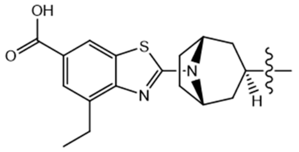
29	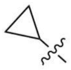	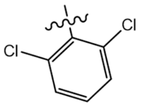	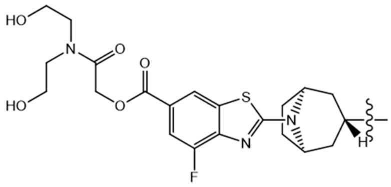
30	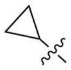	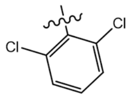	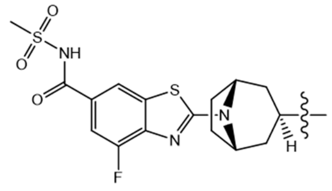
31	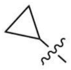	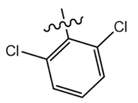	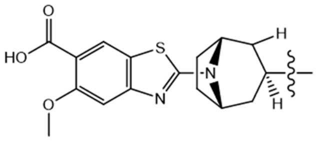
32	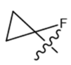	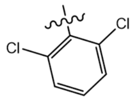	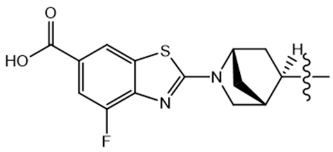
33	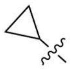	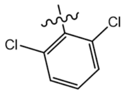	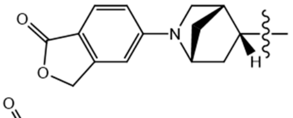
34	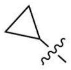	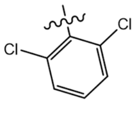	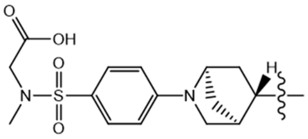

35	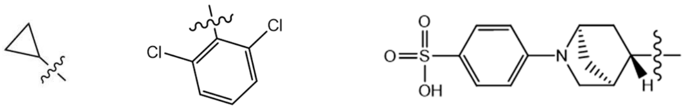
36	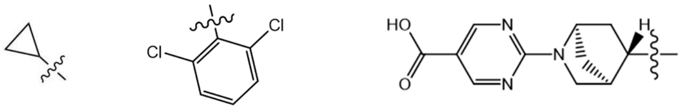
37	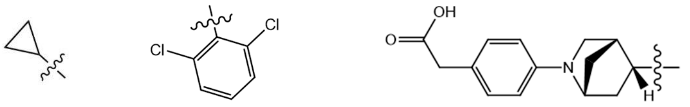
38	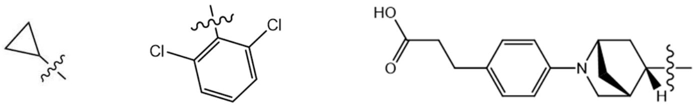
39	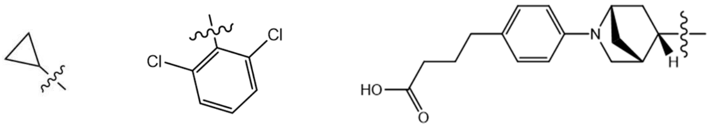
40	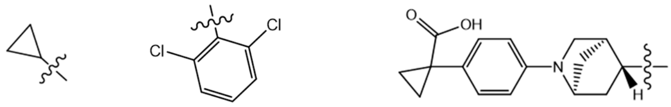
41	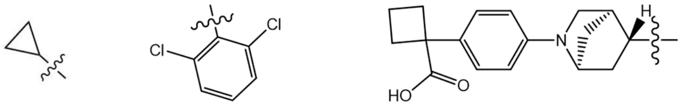
42	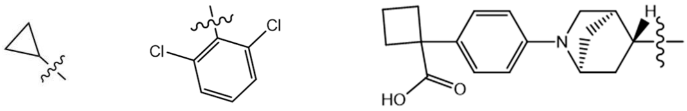
43	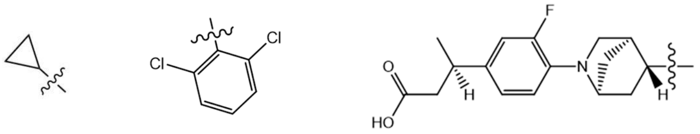
44	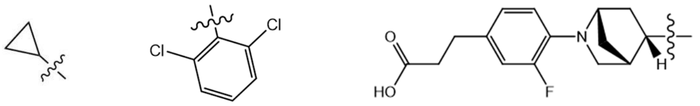
45	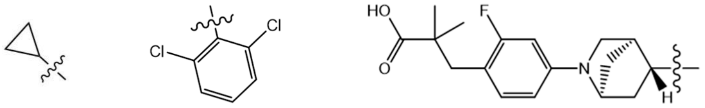
46	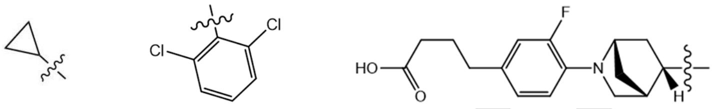
47	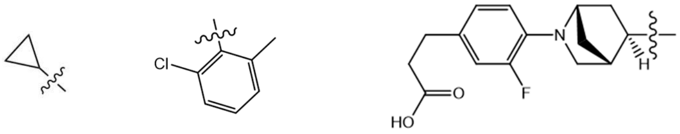
48	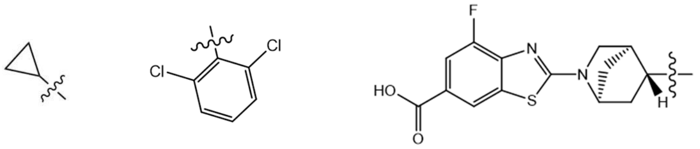
49	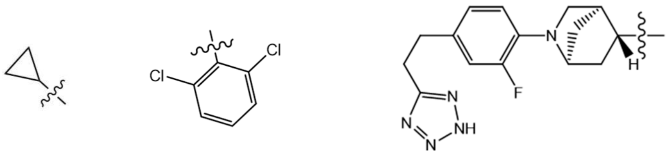

50	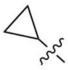	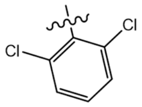	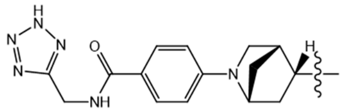
51	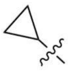	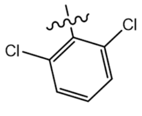	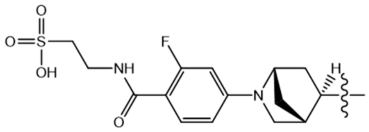
52	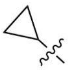	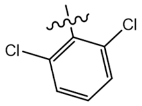	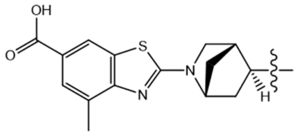
53	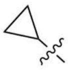	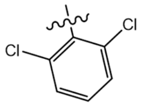	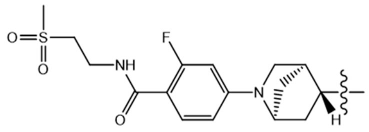
54	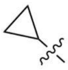	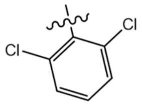	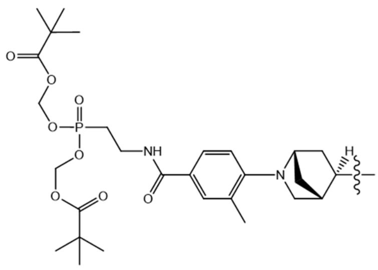
55	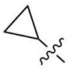	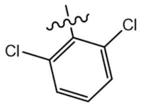	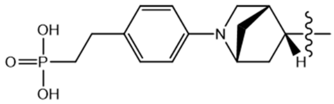
56	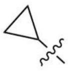	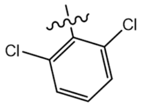	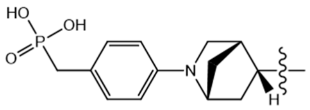
57	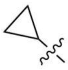	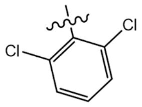	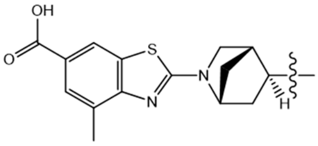
58	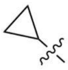	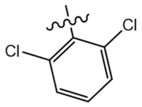	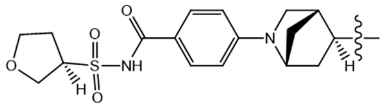
59	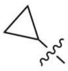	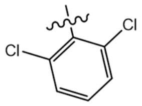	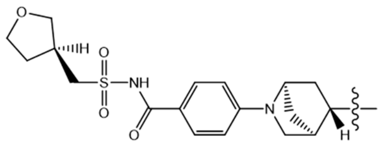
60	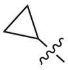	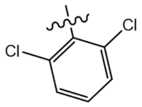	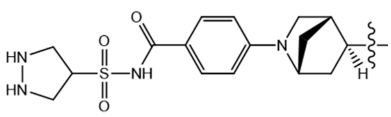
61	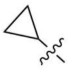	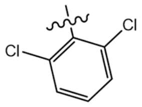	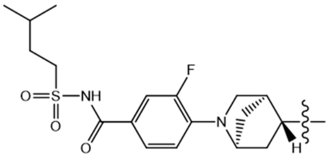

62	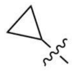	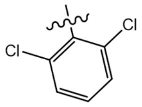	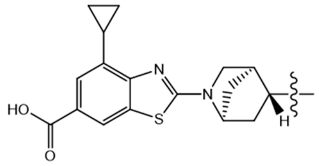
63	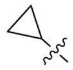	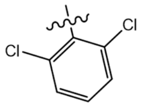	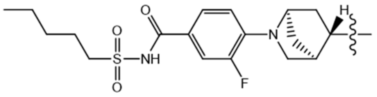
64	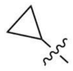	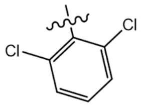	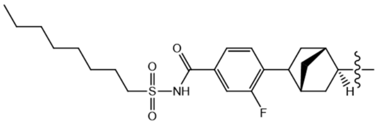
65	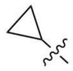	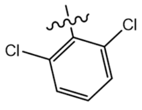	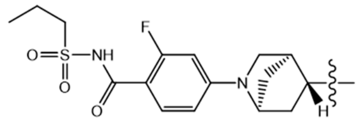
66	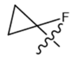	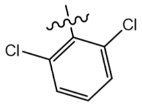	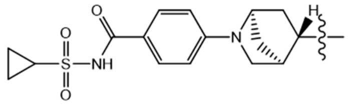
67	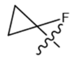	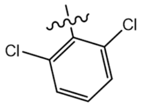	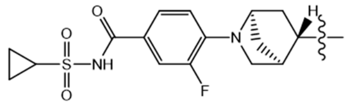
68	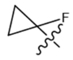	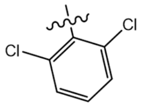	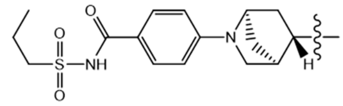
69	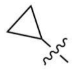	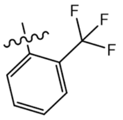	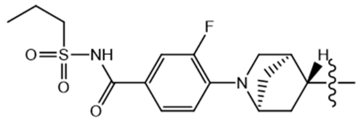
70	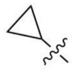	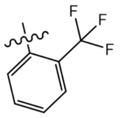	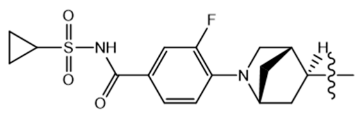
71	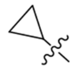	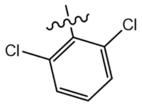	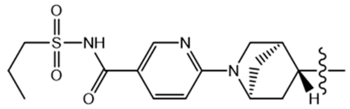
72	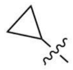	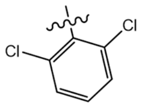	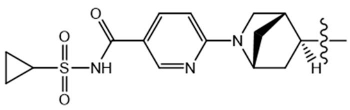
73	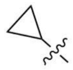	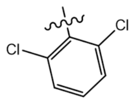	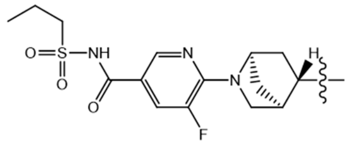

74	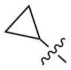	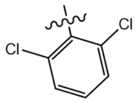	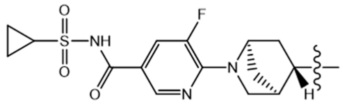
75	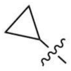	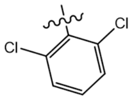	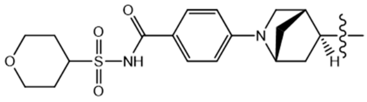
76	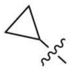	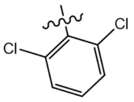	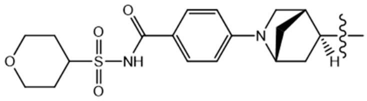
77	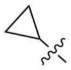	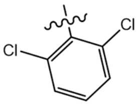	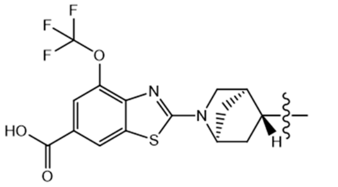
78	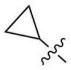	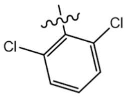	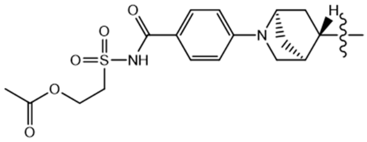
79	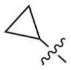	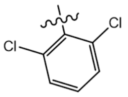	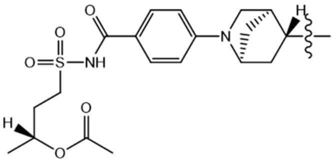
80	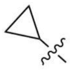	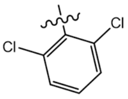	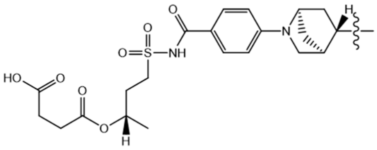
81	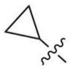	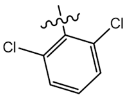	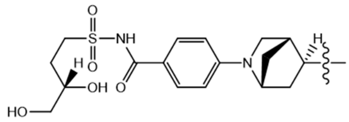
82	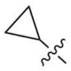	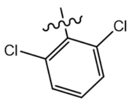	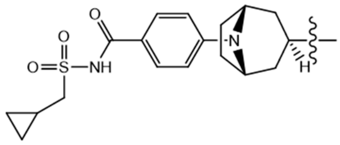
83	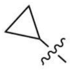	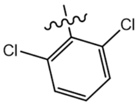	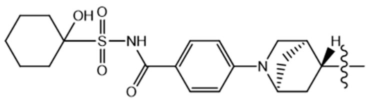
84	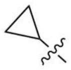	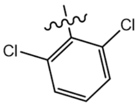	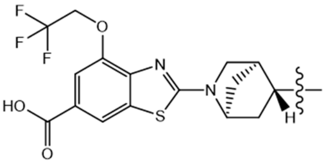

85	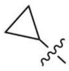	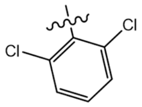	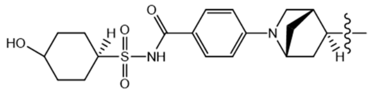
86	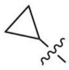	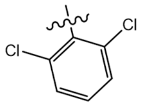	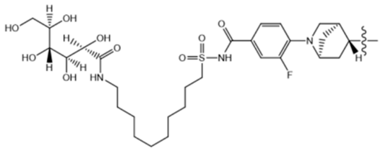
87	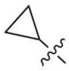	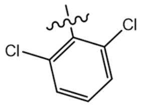	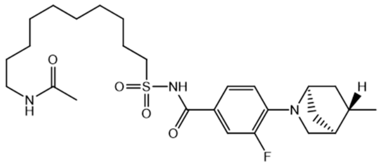
88	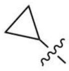	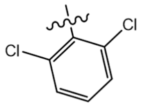	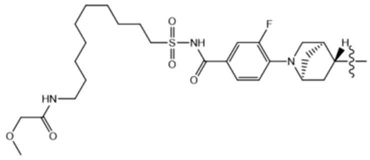
89	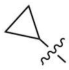	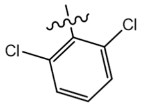	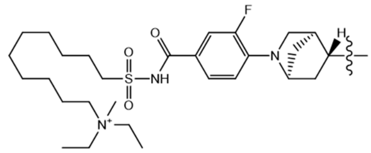
90	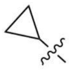	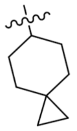	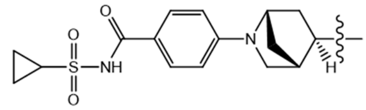
91	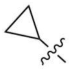	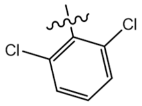	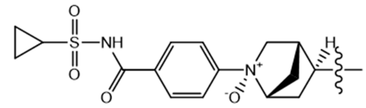
92	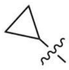	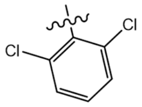	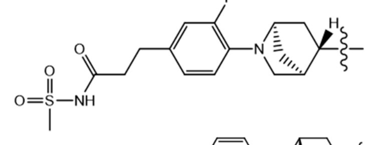
93	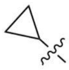	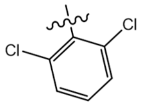	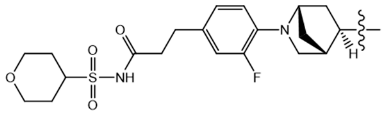
94	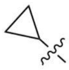	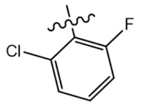	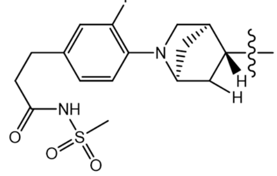
95	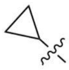	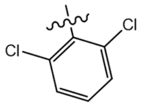	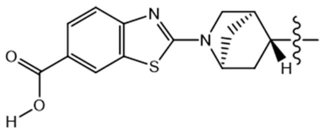

96	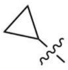	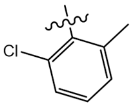	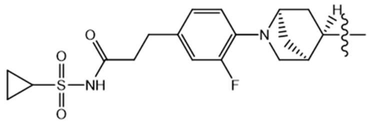
97	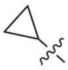	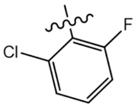	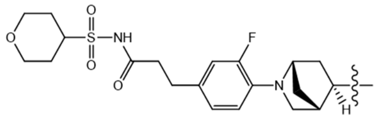
98	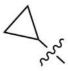	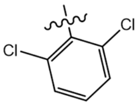	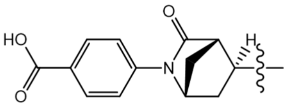
99	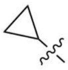	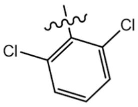	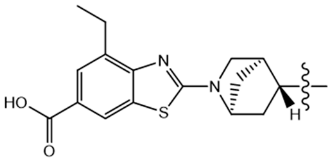
100	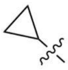	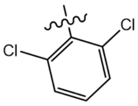	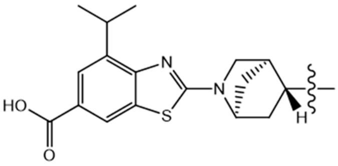
101	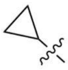	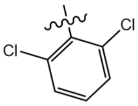	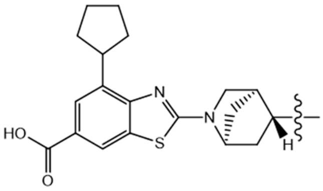
102	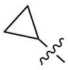	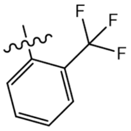	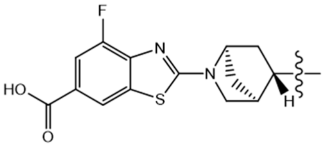
103	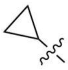	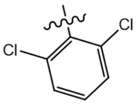	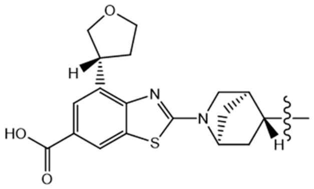
104	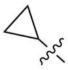	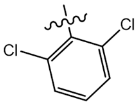	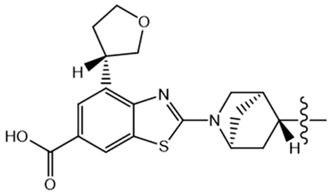
105	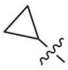	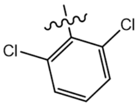	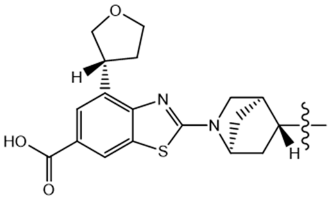
106	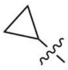	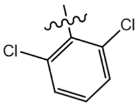	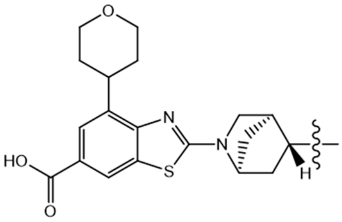

107	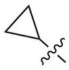	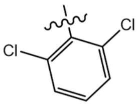	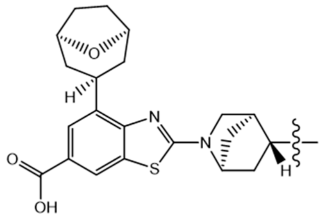
108	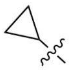	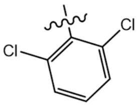	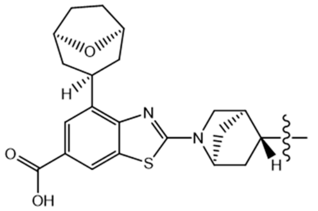
109	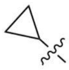	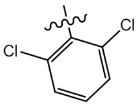	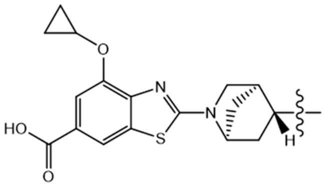
110	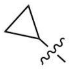	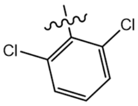	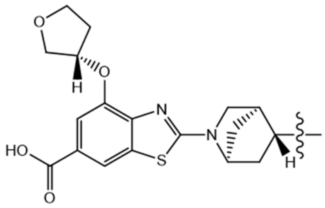
111	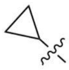	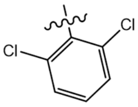	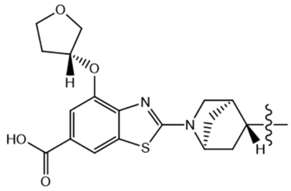
112	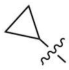	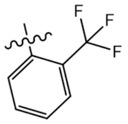	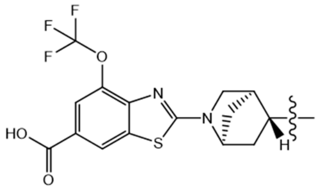
113	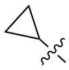	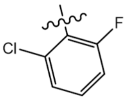	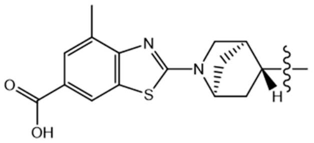
114	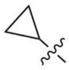	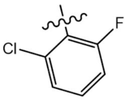	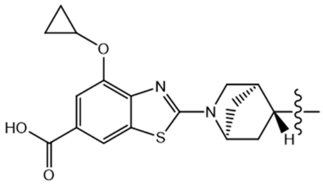
115	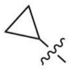	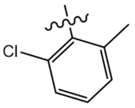	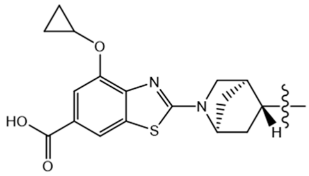

116	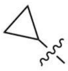	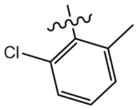	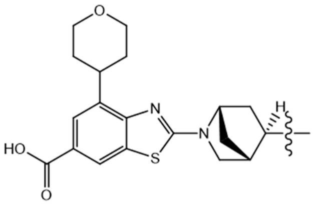
117	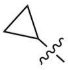	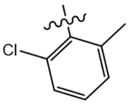	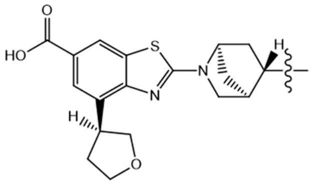
118	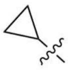	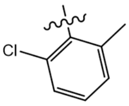	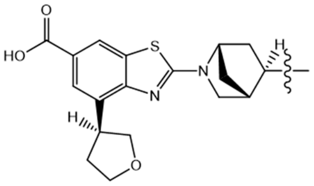
119	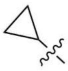	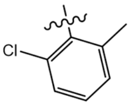	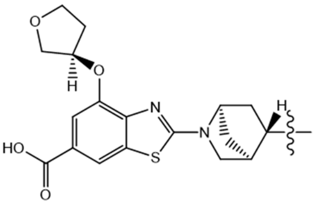
120	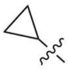	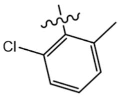	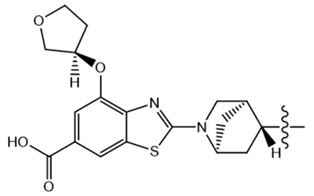
121	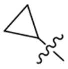	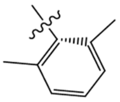	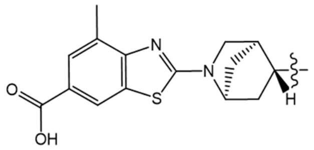
122	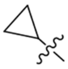	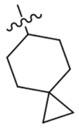	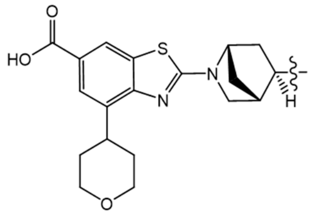
123	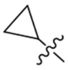	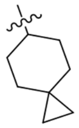	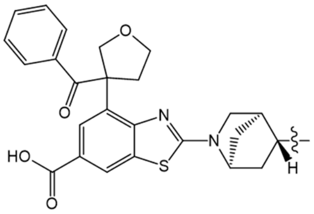
124	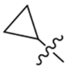	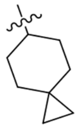	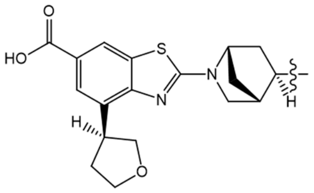

125	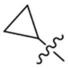	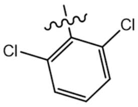	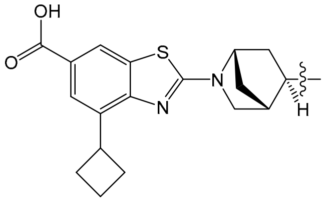
126	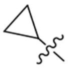	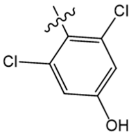	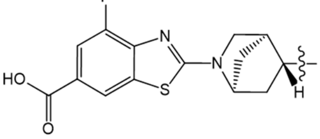
127	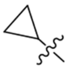	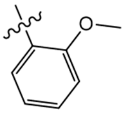	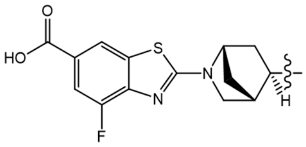
128	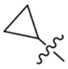	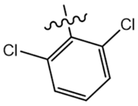	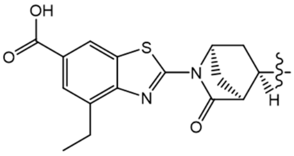
129	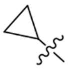	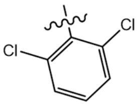	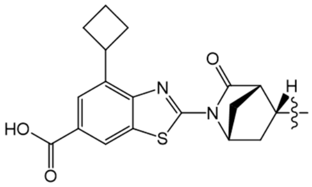
130	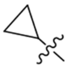	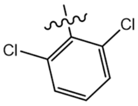	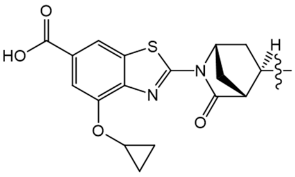
131	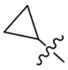	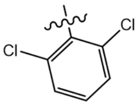	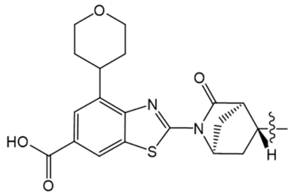
132	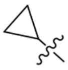	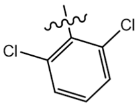	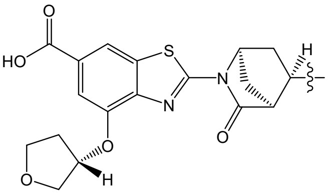
133	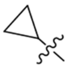	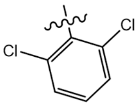	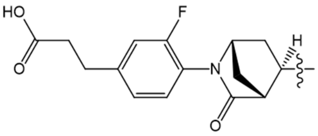
134	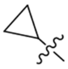	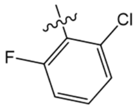	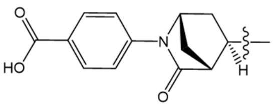

135	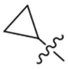	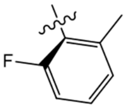	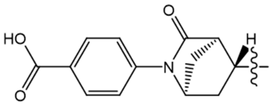
136	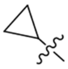	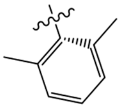	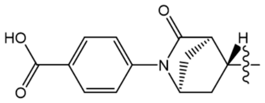
137	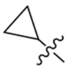	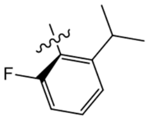	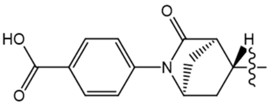
138	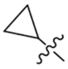	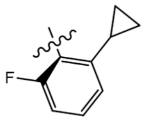	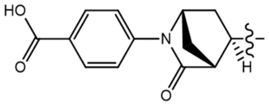
139	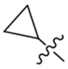	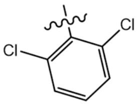	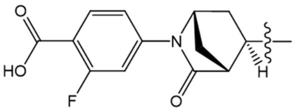
140	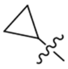	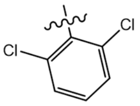	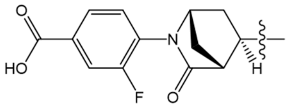
141	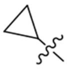	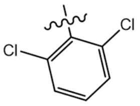	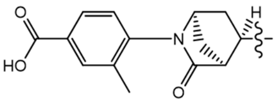
142	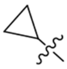	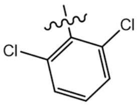	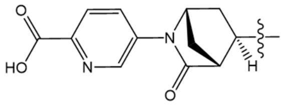
143	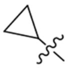	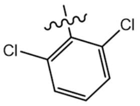	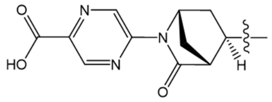
144	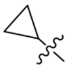	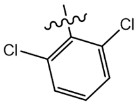	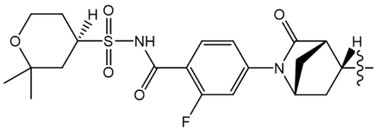
145	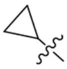	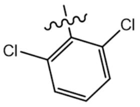	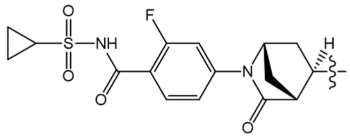
146	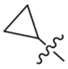	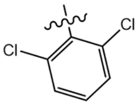	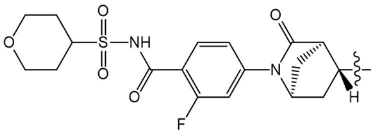
147	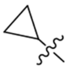	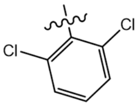	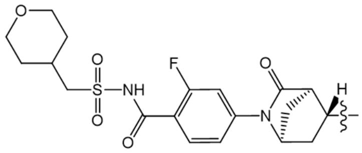

148	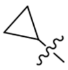	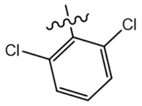	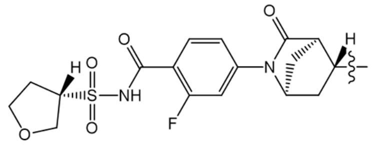
149	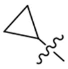	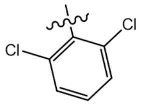	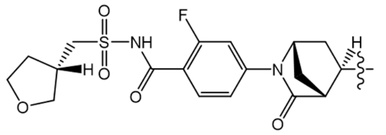
150	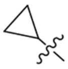	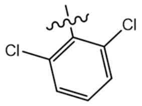	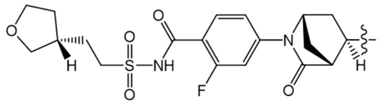
151	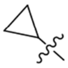	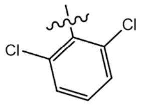	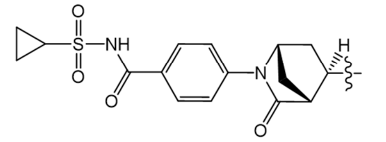
152	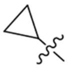	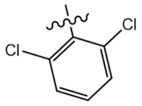	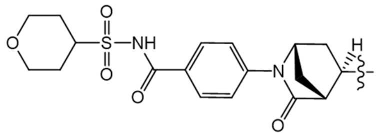
153	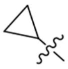	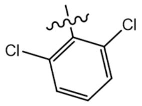	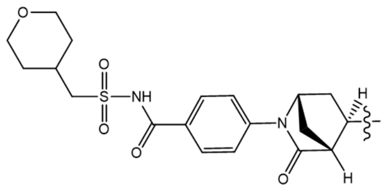
154	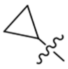	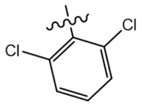	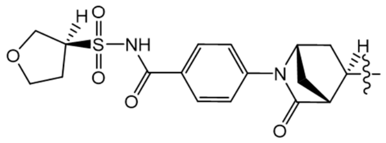
155	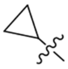	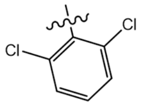	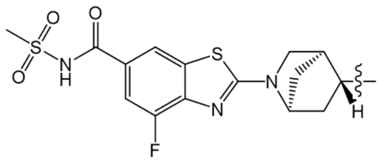
156	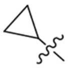	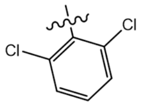	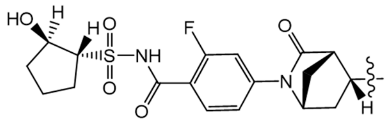
157	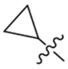	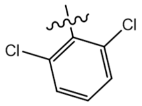	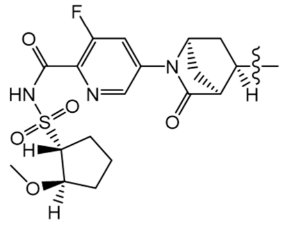
158	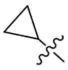	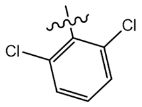	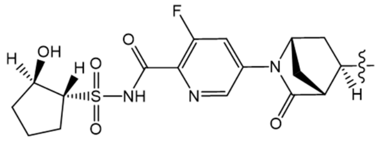
159	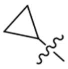	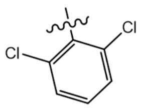	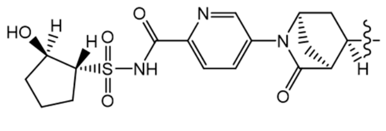

160	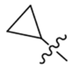	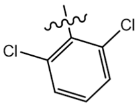	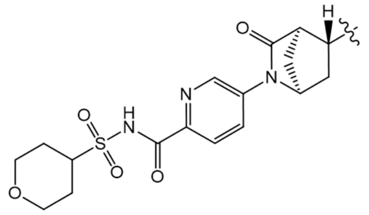
161	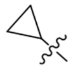	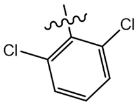	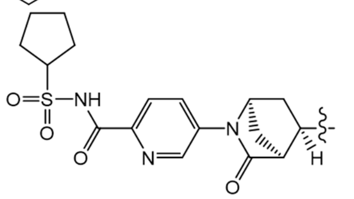
162	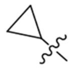	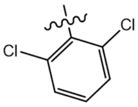	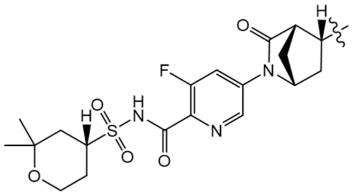
163	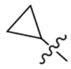	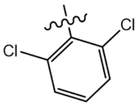	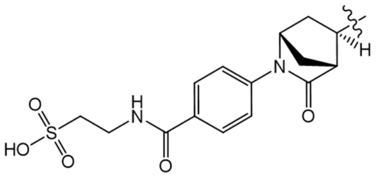
164	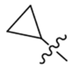	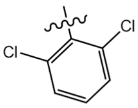	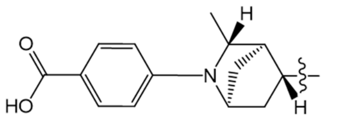
165	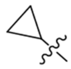	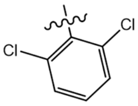	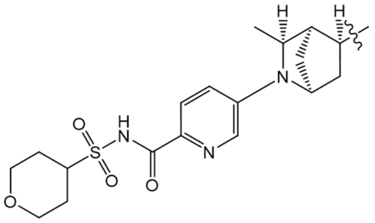
166	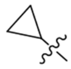	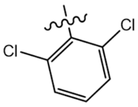	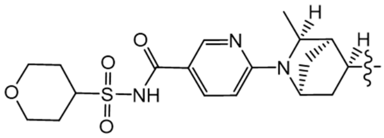
167	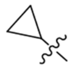	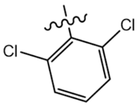	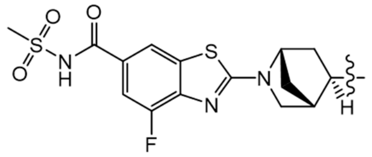
168	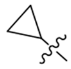	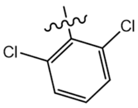	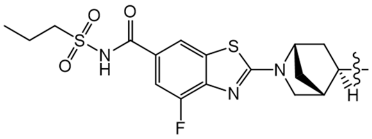
169	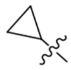	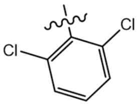	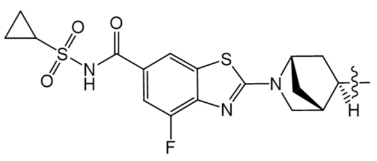

170	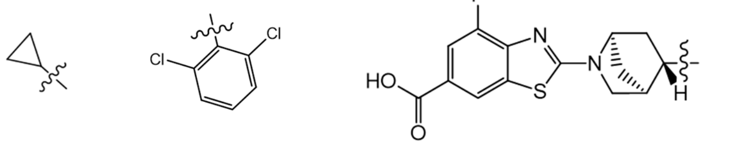
171	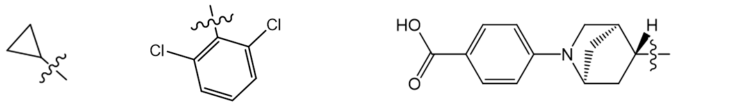
172	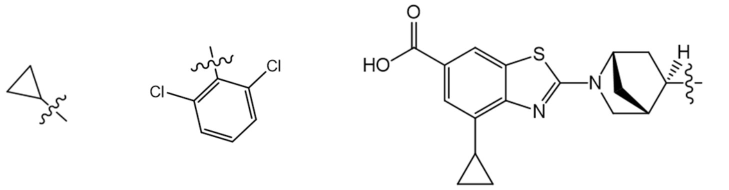
173	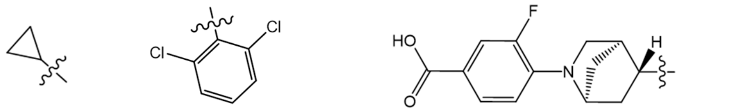
174	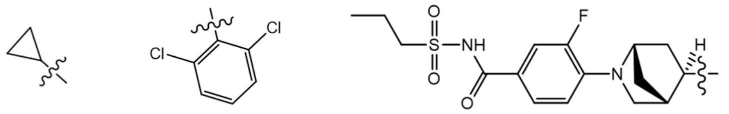
175	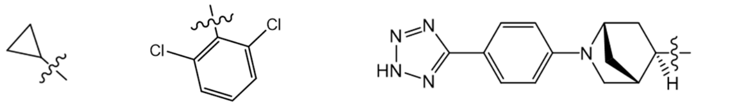
176	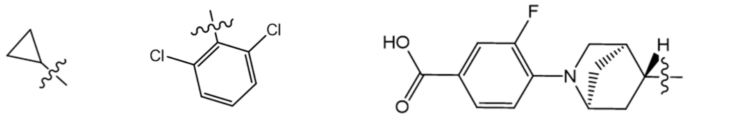
177	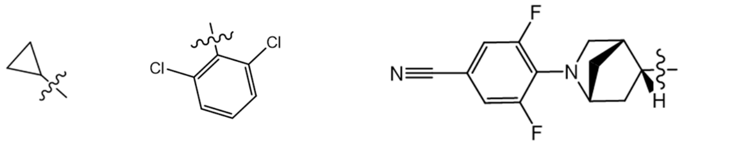
178	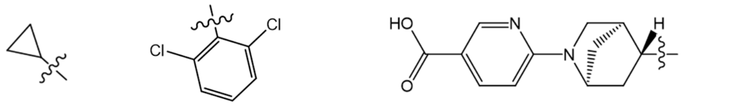
179	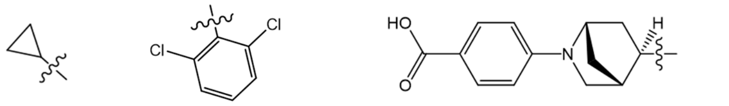
180	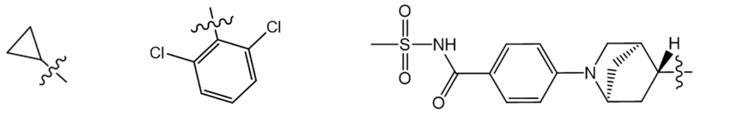
181	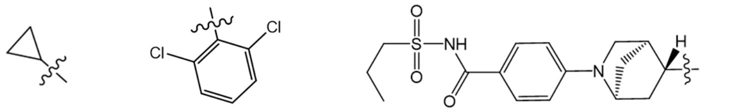
182	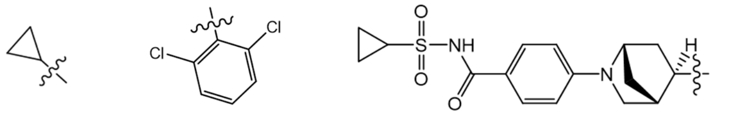
183	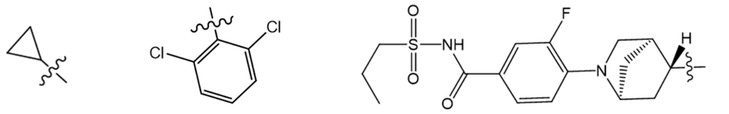
184	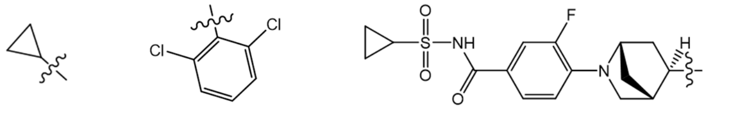

185	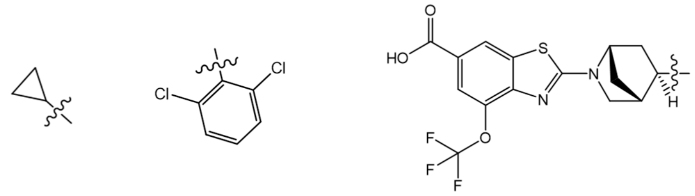
186	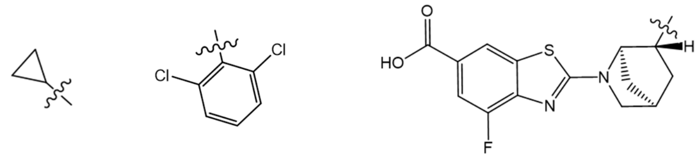
187	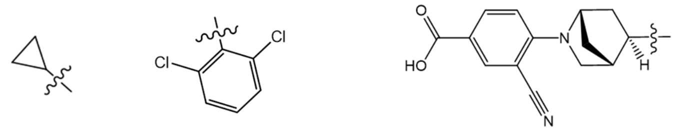
188	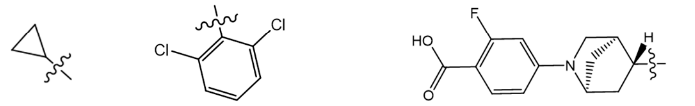
189	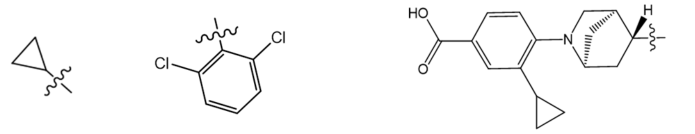
190	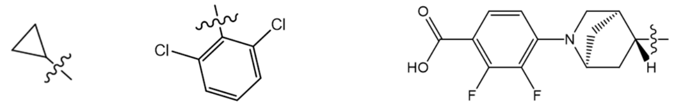
191	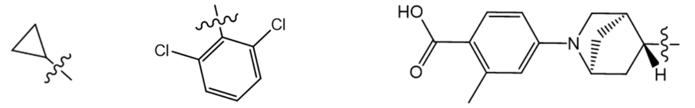
192	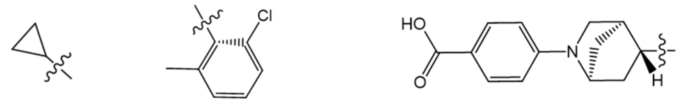
193	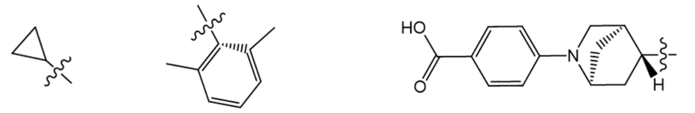
194	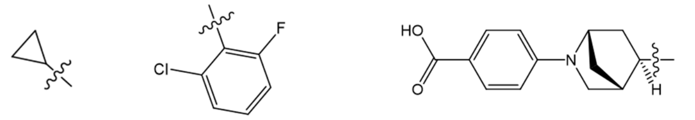
195	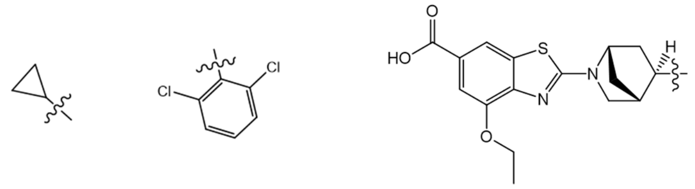
196	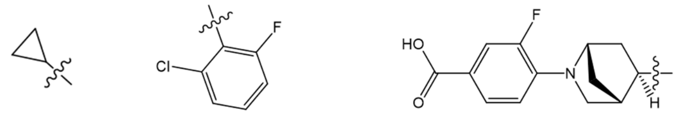
197	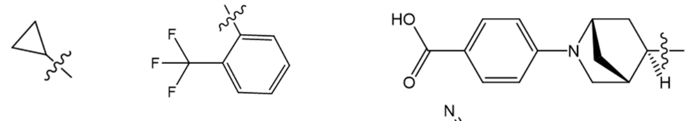
198	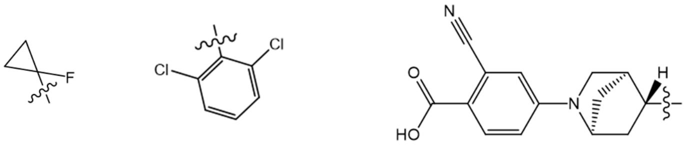

199	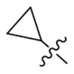	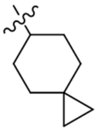	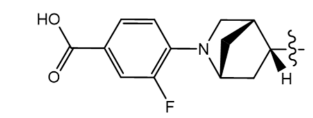
200	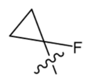	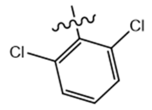	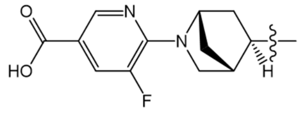
201	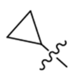	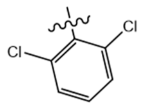	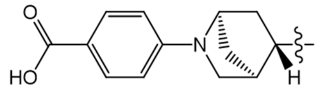
202	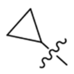	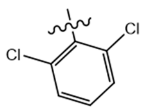	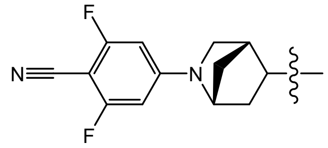
203	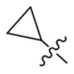	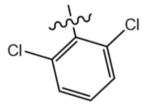	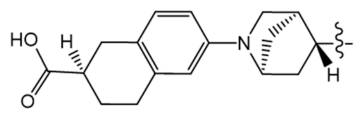
204*	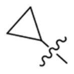	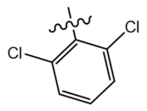	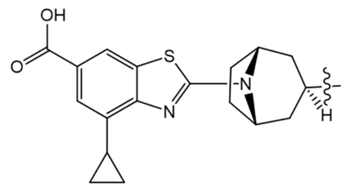
205*	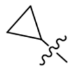	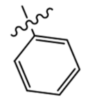	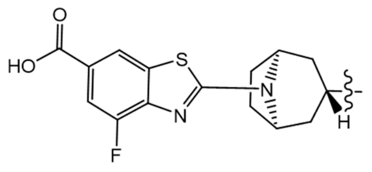
206*	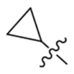	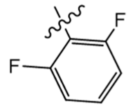	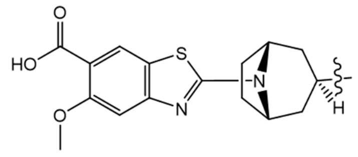
207*	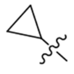	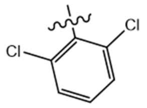	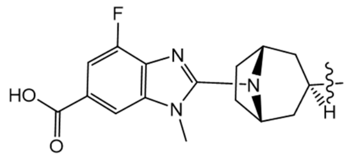
208*	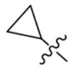	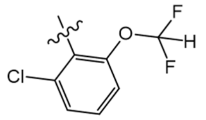	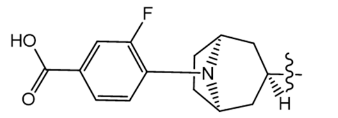
209*	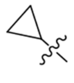	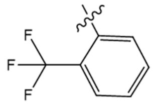	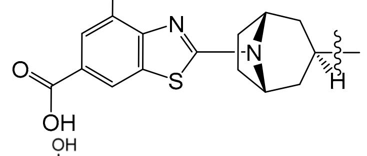
210*	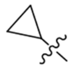	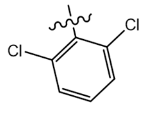	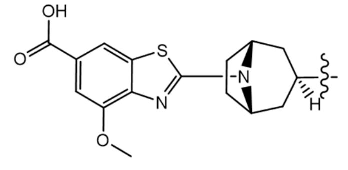

211*	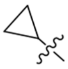	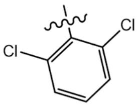	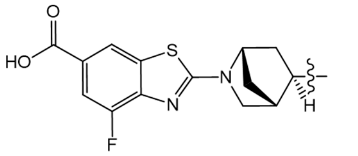
212*	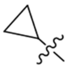	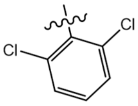	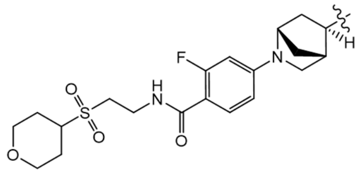
213*	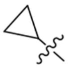	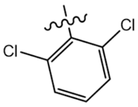	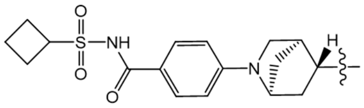
214*	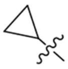	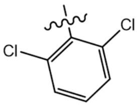	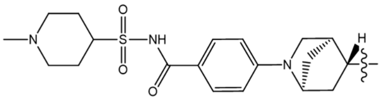
215*	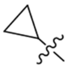	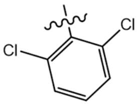	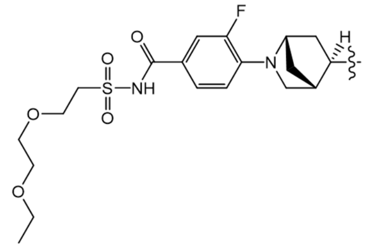
216*	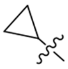	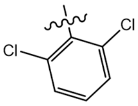	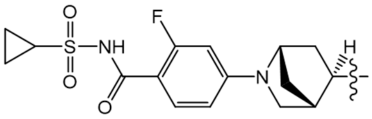
217*	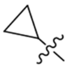	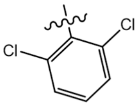	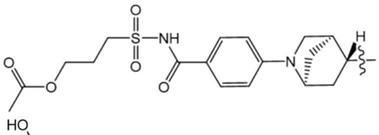
218*	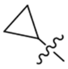	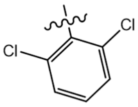	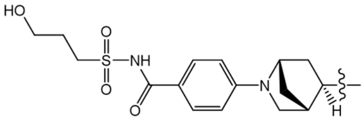
219*	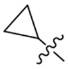	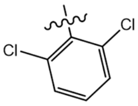	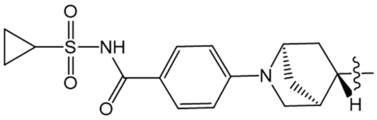
220*	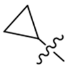	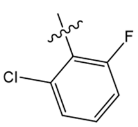	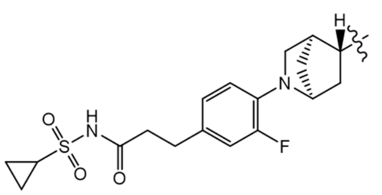
221*	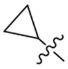	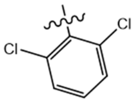	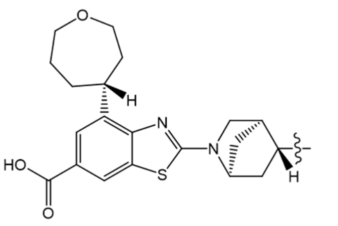

222*	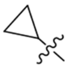	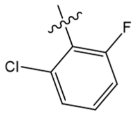	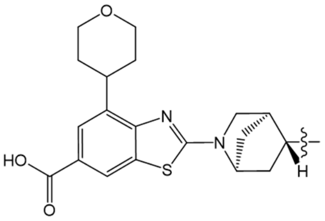
223*	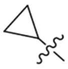	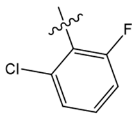	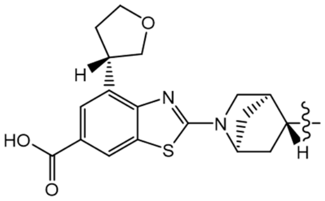
224*	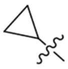	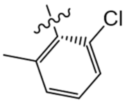	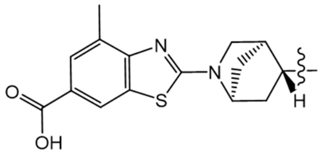
225*	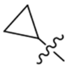	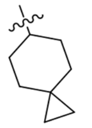	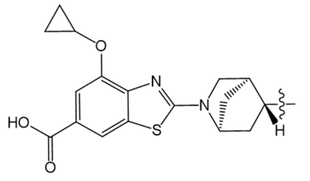
226*	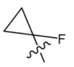	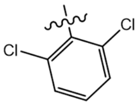	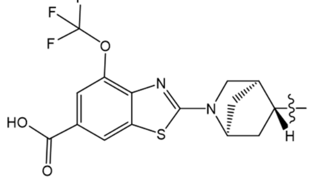
227*	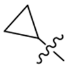	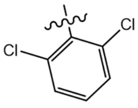	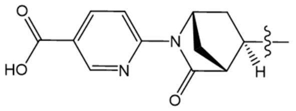
228*	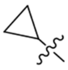	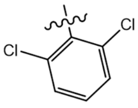	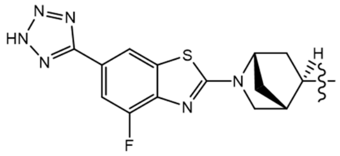
229*	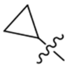	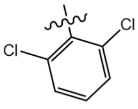	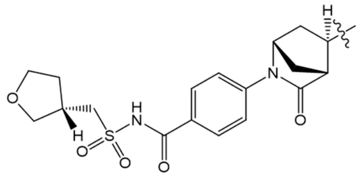
230*	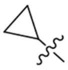	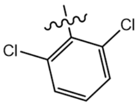	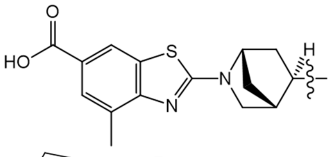
231*	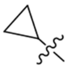	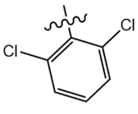	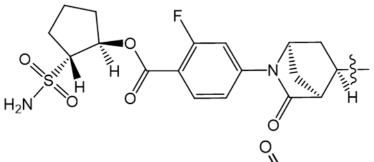
232*	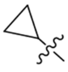	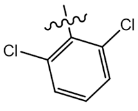	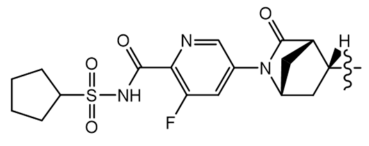

233*	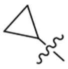	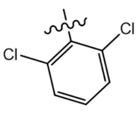	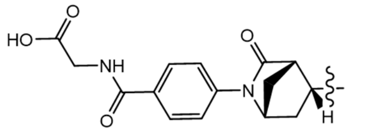
234*	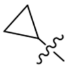	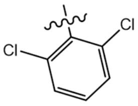	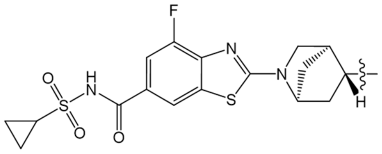
235*	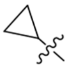	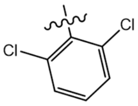	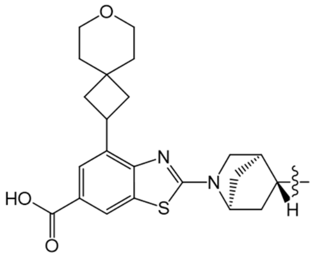
236*	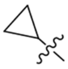	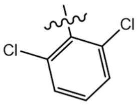	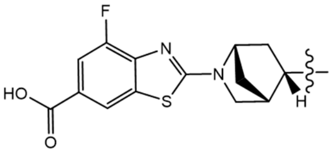
237*	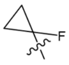	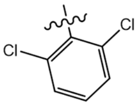	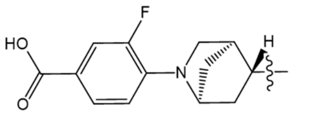
238*	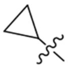	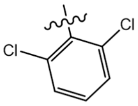	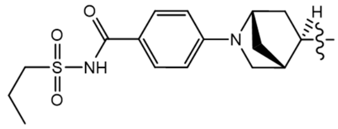
239*	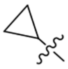	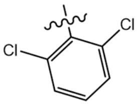	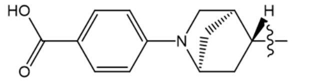
240*	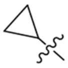	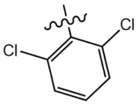	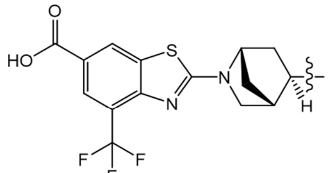
241*	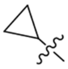	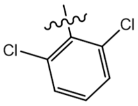	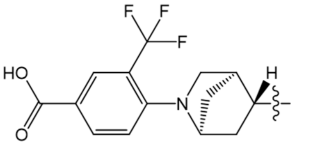
242*	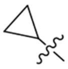	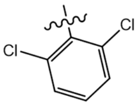	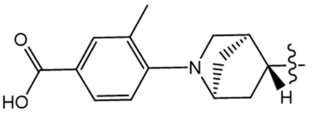
243*	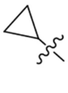	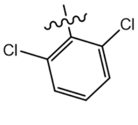	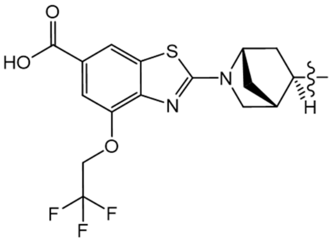

244*	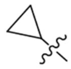	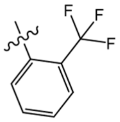	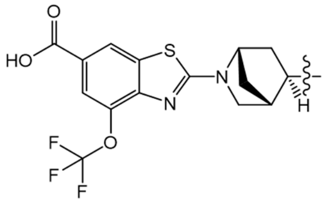
245*	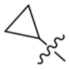	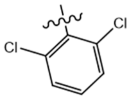	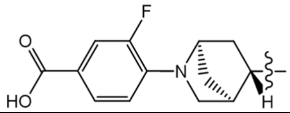

* Test set compounds for the validation of the 3D-QSAR model.

Supplementary Table S2. The actual pEC_{50}, predicted pEC_{50} (Pred.), and the residuals (res.) of dataset compounds.

Compound	pEC_{50}	CoMFA		CoMSIA	
no.	Actual	Pred.	Res.	Pred.	Res.
1	6.5	6.654	-0.154	6.76	-0.26
2	7.4	7.581	-0.181	7.312	0.088
3	7.6	7.461	0.139	7.509	0.091
4	7.4	7.432	-0.032	7.515	-0.115
5	7.4	7.366	0.034	7.257	0.143
6	7.5	7.474	0.026	7.401	0.099
7	7.2	7.192	0.008	7.222	-0.022
8	7.1	7.25	-0.15	7.231	-0.131
9	7.2	7.376	-0.176	7.35	-0.15
10	7.4	7.303	0.097	7.372	0.028
11	6.699	6.545	0.154	6.718	-0.019
12	6.5	6.694	-0.194	6.462	0.038
13	6.6	6.719	-0.119	6.763	-0.163
14	6.9	7.149	-0.249	6.918	-0.018
15	7.6	7.616	-0.016	7.628	-0.028
16	7.3	7.255	0.045	7.356	-0.056
17	6.6	6.475	0.125	6.52	0.08
18	6.801	6.732	0.069	6.684	0.117
19	7.2	7.243	-0.043	7.362	-0.162
20	8.3	8.315	-0.015	8.418	-0.118
21	7.801	7.883	-0.082	7.861	-0.06
22	8	8.088	-0.088	8.001	-0.001
23	7.699	7.482	0.217	7.483	0.216
24	7.3	7.486	-0.186	7.563	-0.263
25	6.2	6.399	-0.199	6.429	-0.229
26	6.6	6.545	0.055	6.623	-0.023
27	7.4	7.392	0.008	7.192	0.208
28	7	7.096	-0.096	7	0
29	6.6	6.623	-0.023	6.639	-0.039
30	7.699	7.736	-0.037	7.859	-0.16
31	7.1	7.015	0.085	6.93	0.17
32	7	7.01	-0.01	7.096	-0.096
33	7	7.175	-0.175	7.141	-0.141
34	8	7.84	0.16	8.066	-0.066
35	8.2	8.08	0.12	8.16	0.04
36	8	7.763	0.237	7.95	0.05
37	7.9	7.885	0.015	7.857	0.043
38	8.1	8.032	0.068	8.081	0.019
39	7.801	8.01	-0.209	8.078	-0.277
40	8.2	8.647	-0.447	8.217	-0.017

41	8.9	8.891	0.009	8.988	-0.088
42	8.6	8.566	0.034	8.618	-0.018
43	8.4	8.365	0.035	8.465	-0.065
44	8.6	8.531	0.069	8.557	0.043
45	7.6	7.777	-0.177	7.58	0.02
46	8.2	7.951	0.249	7.992	0.208
47	8.2	8.213	-0.013	8.206	-0.006
48	9	8.896	0.104	8.794	0.206
49	8.1	8.314	-0.214	8.084	0.016
50	9.699	9.61	0.089	9.727	-0.028
51	8.6	8.36	0.24	8.571	0.029
52	8.6	8.548	0.052	8.549	0.051
53	9.4	9.441	-0.041	9.408	-0.008
54	8.801	8.726	0.075	8.729	0.072
55	8.2	8.216	-0.016	8.219	-0.019
56	7.5	7.628	-0.128	7.515	-0.015
57	8.6	8.548	0.052	8.549	0.051
58	8.9	8.653	0.247	8.644	0.256
59	8.9	8.923	-0.023	8.939	-0.039
60	8.6	8.416	0.184	8.621	-0.021
61	8.4	8.418	-0.018	8.488	-0.088
62	8.6	8.53	0.07	8.556	0.044
63	8.4	8.225	0.175	8.476	-0.076
64	7.9	7.725	0.175	7.736	0.164
65	9	9.177	-0.177	9.06	-0.06
66	8.5	8.453	0.047	8.526	-0.026
67	8.5	8.467	0.033	8.618	-0.118
68	8.5	8.528	-0.028	8.548	-0.048
69	8.6	8.485	0.115	8.468	0.132
70	8.5	8.363	0.137	8.725	-0.225
71	8.4	8.216	0.184	8.22	0.18
72	8.4	8.248	0.152	8.211	0.189
73	8.4	8.419	-0.019	8.49	-0.09
74	8.6	8.589	0.011	8.507	0.093
75	8	8.193	-0.193	8.227	-0.227
76	8.6	8.66	-0.06	8.648	-0.048
77	8.801	8.749	0.052	8.66	0.141
78	8.4	8.319	0.081	8.472	-0.072
79	9.4	9.482	-0.082	9.408	-0.008
80	8.6	8.743	-0.143	8.541	0.059
81	8.6	8.528	0.072	8.625	-0.025
82	8.699	8.755	-0.056	8.776	-0.077
83	8.2	8.552	-0.352	8.536	-0.336
84	8.801	8.747	0.054	8.813	-0.012

85	8.6	8.606	-0.006	8.734	-0.134
86	8.1	8.167	-0.067	8.263	-0.163
87	9.5	9.391	0.109	9.494	0.006
88	9.6	9.707	-0.107	9.562	0.038
89	7	7.172	-0.172	7.133	-0.133
90	8	7.961	0.039	7.897	0.103
91	8.1	8.025	0.075	7.966	0.134
92	8.4	8.409	-0.009	8.385	0.015
93	8.6	8.49	0.11	8.665	-0.065
94	8.2	8.27	-0.07	8.254	-0.054
95	7.3	7.322	-0.022	7.2	0.1
96	8.2	8.267	-0.067	8.175	0.025
97	8.4	8.354	0.046	8.373	0.027
98	8.4	7.876	0.524	7.854	0.546
99	8.5	8.511	-0.011	8.375	0.125
100	8.5	8.539	-0.039	8.621	-0.121
101	7.801	7.747	0.054	7.765	0.036
102	8	7.77	0.23	7.951	0.049
103	9.1	9.017	0.083	9.035	0.065
104	8.6	8.704	-0.104	8.62	-0.02
105	8.6	8.712	-0.112	8.699	-0.099
106	9	8.914	0.086	8.895	0.105
107	8.4	8.396	0.004	8.417	-0.017
108	8.4	8.396	0.004	8.417	-0.017
109	8.5	8.487	0.013	8.577	-0.077
110	8.801	8.673	0.128	8.721	0.08
111	8.6	8.713	-0.113	8.626	-0.026
112	8.4	8.591	-0.191	8.637	-0.237
113	8.6	8.648	-0.048	8.57	0.03
114	8.6	8.58	0.02	8.565	0.035
115	8.699	8.673	0.026	8.598	0.101
116	8.6	8.731	-0.131	8.663	-0.063
117	8.4	8.324	0.076	8.301	0.099
118	8.4	8.341	0.059	8.395	0.005
119	8.801	8.652	0.149	8.802	-0.001
120	8.801	8.742	0.059	8.792	0.009
121	8.2	8.133	0.067	8.253	-0.053
122	7.2	7.272	-0.072	7.14	0.06
123	7.3	7.275	0.025	7.437	-0.137
124	7.4	7.391	0.009	7.397	0.003
125	7.4	7.19	0.21	7.365	0.035
126	9.2	8.934	0.266	9.156	0.044
127	8	7.934	0.066	8.006	-0.006
128	8.2	8.284	-0.084	8.212	-0.012

129	7.6	7.654	-0.054	7.586	0.014
130	9	9.162	-0.162	8.938	0.062
131	7.9	7.984	-0.084	7.833	0.067
132	8.4	8.29	0.11	8.381	0.019
133	6.6	6.431	0.169	6.589	0.011
134	8.2	7.895	0.305	7.97	0.23
135	7.4	7.39	0.01	7.61	-0.21
136	7.6	7.543	0.057	7.61	-0.01
137	7.6	7.546	0.054	7.406	0.194
138	7.801	7.912	-0.111	7.836	-0.035
139	8.4	7.987	0.413	8.221	0.179
140	7.4	7.687	-0.287	7.912	-0.512
141	6.699	6.711	-0.012	6.724	-0.025
142	7.6	7.905	-0.305	7.674	-0.074
143	7.1	7.62	-0.52	7.382	-0.282
144	8.801	8.734	0.067	8.823	-0.022
145	8.699	8.837	-0.138	8.575	0.124
146	8.801	8.832	-0.031	8.88	-0.079
147	8.801	8.825	-0.024	8.732	0.069
148	9	8.993	0.007	8.93	0.07
149	8.801	8.881	-0.08	8.801	0
150	8.6	8.498	0.102	8.596	0.004
151	8.6	8.685	-0.085	8.529	0.071
152	8.9	8.875	0.025	8.897	0.003
153	8.6	8.681	-0.081	8.513	0.087
154	8.801	8.922	-0.121	8.96	-0.159
155	8.9	8.763	0.137	8.863	0.037
156	8.2	8.312	-0.112	8.167	0.033
157	8	8.093	-0.093	7.948	0.052
158	8.2	8.216	-0.016	8.128	0.072
159	8.1	8.275	-0.175	8.042	0.058
160	8	8.105	-0.105	8.04	-0.04
161	8.3	8.362	-0.062	8.334	-0.034
162	8.4	8.452	-0.052	8.337	0.063
163	8.4	8.37	0.03	8.367	0.033
164	8.6	8.586	0.014	8.678	-0.078
165	6.1	6.045	0.055	6.078	0.022
166	6	5.976	0.024	5.94	0.06
167	7.4	7.423	-0.023	7.439	-0.039
168	7.6	7.442	0.158	7.42	0.18
169	7.6	7.659	-0.059	7.645	-0.045
170	6.3	6.276	0.024	6.219	0.081
171	7.6	7.763	-0.163	7.77	-0.17
172	7.3	7.437	-0.137	7.415	-0.115

173*	7.801	7.713	0.088	7.822	-0.021
174	8	7.941	0.059	8.05	-0.05
175	6.801	7.034	-0.233	6.777	0.024
176	8.699	8.592	0.107	8.576	0.123
177	7	7.059	-0.059	7.084	-0.084
178	7.9	8.293	-0.393	8.239	-0.339
179	8.5	8.374	0.126	8.35	0.15
180	8.4	8.587	-0.187	8.563	-0.163
181	8.2	8.344	-0.144	8.152	0.048
182	8.2	8.407	-0.207	8.561	-0.361
183	8.699	8.434	0.265	8.745	-0.046
184	7.9	8.137	-0.237	8.169	-0.269
185	7.699	7.316	0.383	7.382	0.317
186	6.9	6.812	0.088	6.794	0.106
187	8.6	8.843	-0.243	8.773	-0.173
188	8.699	8.435	0.264	8.507	0.192
189	9.1	9.162	-0.062	9.124	-0.024
190	10	9.607	0.393	9.778	0.222
191	9.3	9.263	0.037	8.962	0.338
192	8.4	8.252	0.148	8.224	0.176
193	8.2	8.174	0.026	8.233	-0.033
194	8.6	8.632	-0.032	8.509	0.091
195	7.6	7.404	0.196	7.43	0.17
196	8.699	8.609	0.09	8.57	0.129
197	8	8.18	-0.18	8.054	-0.054
198	8.6	8.859	-0.259	8.744	-0.144
199	7.1	7.014	0.086	7.054	0.046
200	8	7.863	0.137	7.986	0.014
201	7.3	7.425	-0.125	7.368	-0.068
202	7.4	7.233	0.167	7.142	0.258
203	8	7.922	0.078	7.906	0.094
204*	7.4	7.2492	0.1508	7.1959	0.2041
205*	6.699	7.0211	-0.3221	7.2052	-0.5062
206*	6.699	6.897	-0.198	7.1045	-0.4055
207*	6.6	6.9799	-0.3799	7.0873	-0.4873
208*	7.3	7.1561	0.1439	7.0996	0.2004
209*	7.1	7.1255	-0.0255	7.1307	-0.0307
210*	8	7.9199	0.0801	7.8217	0.1783
211*	7.4	7.1849	0.2151	7.2773	0.1227
212*	8.4	8.3495	0.0505	8.8815	-0.4815
213*	8.6	8.5289	0.0711	8.8723	-0.2723
214*	9.2	9.239	-0.039	9.4207	-0.2207
215*	8.2	8.2095	-0.0095	7.9153	0.2847
216*	8.801	8.3912	0.4098	8.4707	0.3303

217*	8.6	8.5964	0.0036	8.8511	-0.2511
218*	8.6	8.6114	-0.0114	8.5658	0.0342
219*	8.1	7.7819	0.3181	8.3831	-0.2831
220*	8.1	7.6584	0.4416	7.9346	0.1654
221*	8.6	8.7142	-0.1142	8.759	-0.159
222*	8.5	8.8709	-0.3709	8.8203	-0.3203
223*	8.6	8.6187	-0.0187	8.406	0.194
224*	8.5	8.1158	0.3842	8.1323	0.3677
225*	7.699	7.6051	0.0939	7.7469	-0.0479
226*	8.4	8.6122	-0.2122	8.5629	-0.1629
227*	7.4	7.786	-0.386	7.5074	-0.1074
228*	8.4	8.3879	0.0121	8.3732	0.0268
229*	8.699	8.3484	0.3506	8.5301	0.1689
230*	7.6	7.4653	0.1347	7.318	0.282
231*	8.5	8.0472	0.4528	8.356	0.144
232*	8.6	8.6236	-0.0236	8.4201	0.1799
233*	8	8.0466	-0.0466	8.0473	-0.0473
234*	8.6	8.5592	0.0408	8.6489	-0.0489
235*	8.6	8.4048	0.1952	8.5463	0.0537
236*	7.801	7.6695	0.1315	7.6583	0.1427
237*	7.6	7.8658	-0.2658	7.5385	0.0615
238*	7.9	7.6984	0.2016	7.6894	0.2106
239*	8.801	8.4824	0.3186	8.5161	0.2849
240*	7.2	6.8457	0.3543	7.1862	0.0138
241*	8.6	8.8797	-0.2797	8.3435	0.2565
242*	8.6	8.9032	-0.3032	8.684	-0.084
243*	7.4	7.3286	0.0714	7.3645	0.0355
244*	7.1	7.0478	0.0522	7.4286	-0.3286
245*	8	7.8382	0.1618	7.7444	0.2556

* Test set compounds for the validation of the 3D-QSAR model.
